# Supplementary material for: Effect of a perinatal care quality improvement package on patient satisfaction: a secondary outcome analysis of a cluster-randomised controlled trial
Source: BMJ Open. 2022 Jun 6;12(6):e054544. doi: 10.1136/bmjopen-2021-054544 (PMC9171223; doi:10.1136/bmjopen-2021-054544)
Supplement: Supplementary data [file bmjopen-2021-054544supp001.pdf]

Table S1. Binary logistic regression of patient satisfaction and quality measures of experience of care, by hospital. Adjusted for caste/ethnicity and education level (n=54,919).

		Satisfaction			Informed			Educated			Privacy			Abuse			Respect			Companion		
**Hospital**		Yes N(%)	No N(%)	Adj OR (95% CI)	Yes N(%)	No N(%)	Adj OR (95% CI)	Yes N(%)	No N(%)	Adj OR (95% CI)	Yes N(%)	No N(%)	Adj OR (95% CI)	Yes N(%)	No N(%)	AdjOR (95% CI)	Yes N(%)	No N(%)	AdjOR (95% CI)	Yes N(%)	No N(%)	AdjOR (95% CI)
Mid Western Region	Control	194 (66.4)	98 (33.6)	Ref	284 (97.3)	8 (2.7)	Ref	27 (9.2)	265 (90.8)	Ref	20 (6.8)	272 (93.2)	Ref	1 (0.3)	291 (99.7)	Ref	290 (99.3)	2 (0.7)	Ref	2 (0.7)	290 (99.3)	Ref
	Intervention	2318 (54.1)	1966 (45.9)	0.59 (0.46-0.76)	4252 (99.3)	30 (0.7)	3.99 (1.81-8.80)	1525 (35.6)	2759 (64.4)	5.45 (3.65-8.14)	1171 (27.3)	3113 (72.7)	5.12 (3.24-8.11)	42 (1.0)	4242 (99.0)	2.81 (0.39-20.5)	4259 (99.4)	25 (0.6)	1.14 (0.27-4.84)	12 (0.3)	4270 (99.7)	0.38 (0.08-1.70)
Bharatpur	Control	751 (23.7)	2418 (76.3)	Ref	2945 (93.7)	198 (6.3)	Ref	863 (27.2)	2306 (72.8)	Ref	871 (27.5)	2298 (72.5)	Ref	102 (3.2)	3067 (96.8)	Ref	3057 (96.5)	112 (3.5)	Ref	1285 (40.9)	1858 (59.1)	Ref
	Intervention	4971 (52.3)	4527 (47.7)	3.57 (3.26-3.91)	9224 (97.2)	265 (2.8)	2.34 (1.94-2.83)	5708 (60.1)	3790 (39.9)	4.07 (3.72-4.45)	5091 (53.6)	4407 (46.4)	3.08 (2.82-3.37)	63 (0.7)	9435 (99.3)	0.20 (0.15-0.27)	9421 (99.2)	77 (0.8)	4.54 (3.39-6.09)	174 (1.8)	9315 (98.2)	0.03 (0.02-0.03)
Seti Zonal	Control	919 (41.3)	1304 (58.7)	Ref	1959 (88.2)	261 (11.8)	Ref	421 (18.9)	1802 (81.1)	Ref	867 (39.0)	1356 (61.0)	Ref	37 (1.7)	2186 (98.3)	Ref	2180 (98.1)	43 (1.9)	Ref	1623 (73.1)	597 (26.9)	Ref
	Intervention	3778 (58.9)	2636 (41.1)	2.07 (1.87-2.28)	6107 (95.3)	303 (4.7)	2.74 (2.30-3.26)	3056 (47.6)	3358 (52.4)	3.93 (3.50-4.42)	3658 (57.0)	2756 (43.0)	2.11 (1.92-2.33)	35 (0.5)	6379 (99.5)	0.32 (0.20-0.50)	6331 (98.7)	83 (1.3)	1.53 (1.05-2.21)	5814 (90.7)	596 (9.3)	3.59 (3.16-4.07)
Koshi Zonal	Control	2777 (85.6)	468 (14.4)	Ref	2190 (67.8)	1040 (32.2)	Ref	2327 (71.7)	918 (28.3)	Ref	2606 (80.3)	639 (19.7)	Ref	9 (0.3)	3236 (99.7)	Ref	3219 (99.2)	26 (0.8)	Ref	495 (15.3)	2735 (84.7)	Ref
	Intervention	3886 (90.7)	398 (9.3)	1.83 (1.57-2.12)	3148 (73.7)	1124 (26.3)	1.33 (1.20-1.47)	2841 (66.3)	1443 (33.7)	0.82 (0.74-0.90)	2859 (66.7)	1425 (33.3)	0.53 (0.48-0.60)	32 (0.7)	4252 (99.3)	2.59 (1.21-5.53)	4252 (99.3)	32 (0.7)	0.99 (0.58-1.69)	256 (6.0)	4016 (94.0)	0.36 (0.31-0.43)

Rapti Subregional	Control	785 (31.8)	1686 (68.2)	Ref	995 (40.3)	1473 (59.7)	Ref	569 (23.0)	1902 (77.0)	Ref	1108 (44.8)	1363 (55.2)	Ref	17 (0.7)	2454 (99.3)	Ref	2424 (98.1)	47 (1.9)	Ref	22 (0.9)	2446 (99.1)	Ref
	Intervention	271 (15.5)	1473 (84.5)	0.39 (0.34-0.46)	266 (15.3)	1478 (84.7)	0.27 (0.23-0.31)	96 (5.5)	1648 (94.5)	0.19 (0.15-0.24)	875 (50.2)	869 (49.8)	1.23 (1.09-1.39)	17 (1.0)	1727 (99.0)	1.42 (0.72-2.80)	1732 (99.3)	12 (0.7)	2.86 (1.51-5.42)	29 (1.7)	1715 (98.3)	1.97 (1.13-3.46)
Lumbini zonal	Control	5512 (87.5)	791 (12.5)	Ref	4445 (71.0)	1813 (29.0)	Ref	4032 (64.0)	2271 (36.0)	Ref	4378 (69.5)	1925 (30.5)	Ref	73 (1.2)	6230 (98.8)	Ref	6268 (99.4)	35 (0.6)	Ref	183 (2.9)	6075 (97.1)	Ref
	Intervention	4242 (88.3)	564 (11.7)	1.05 (0.94-1.18)	2739 (57.1)	2058 (42.9)	0.54 (0.50-0.58)	2830 (58.9)	1976 (41.1)	0.80 (0.74-0.87)	2987 (62.2)	1819 (37.8)	0.72 (0.66-0.78)	17 (0.4)	4789 (99.6)	0.31 (0.18-0.52)	4764 (99.1)	42 (0.9)	0.63 (0.40-0.98)	42 (0.9)	4755 (99.1)	0.30 (0.21-0.42)
Bheri Zonal	Control	1547 (40.2)	2305 (59.8)	Ref	3621 (94.1)	228 (5.9)	Ref	1089 (28.3)	2763 (71.7)	Ref	1272 (33.0)	2580 (67.0)	Ref	8 (0.2)	3844 (99.8)	Ref	3842 (99.7)	10 (0.3)	Ref	13 (0.3)	3836 (99.7)	Ref
	Intervention	1204 (51.6)	1130 (48.4)	1.62 (1.46-1.80)	1146 (49.3)	1179 (50.7)	0.61 (0.05-0.07)	386 (16.5)	1948 (83.5)	0.51 (0.45-0.58)	373 (16.0)	1961 (84.0)	0.39 (0.34-0.45)	9 (0.4)	2325 (99.6)	1.88 (0.72-4.87)	2315 (99.2)	19 (0.8)	0.32 (0.15-0.69)	1 (0.0)	2324 (100.0)	0.13 (0.02-0.96)
All	Control	12485 (57.9)	9070 (42.1)	Ref	16439 (76.6)	5021 (23.4)	Ref	9328 (43.3)	12227 (56.7)	Ref	11122 (51.6)	10433 (48.4)	Ref	247 (1.1)	21308 (98.9)	Ref	21280 (98.7)	275 (1.3)	Ref	3623 (16.9)	17837 (83.1)	Ref
	Intervention	20670 (62.0)	12694 (38.0)	1.20 (1.16-1.25)	26882 (80.7)	6437 (19.3)	1.26 (1.21-1.31)	16442 (49.3)	16922 (50.7)	1.28 (1.24-1.33)	17014 (51.0)	16350 (49.0)	0.98 (0.95-1.01)	215 (0.6)	33149 (99.4)	0.55 (0.46-0.66)	33074 (99.1)	290 (0.9)	1.49 (1.27-1.76)	6328 (19.0)	26991 (81.0)	1.12 (1.07-1.17)
