# Supplementary material for: Effect of a perinatal care quality improvement package on patient satisfaction: a secondary outcome analysis of a cluster-randomised controlled trial
Source: BMJ Open. 2022 Jun 6;12(6):e054544. doi: 10.1136/bmjopen-2021-054544 (PMC9171223; doi:10.1136/bmjopen-2021-054544)
Supplement: Author's manuscript [file bmjopen-2021-054544.draft_revisions.pdf]

BMJ Open is committed to open peer review. As part of this commitment we make the peer review history of every article we publish publicly available.

When an article is published we post the peer reviewers' comments and the authors' responses online. We also post the versions of the paper that were used during peer review. These are the versions that the peer review comments apply to.

The versions of the paper that follow are the versions that were submitted during the peer review process. They are not the versions of record or the final published versions. They should not be cited or distributed as the published version of this manuscript.

BMJ Open is an open access journal and the full, final, typeset and author-corrected version of record of the manuscript is available on our site with no access controls, subscription charges or pay-per-view fees (http://bmjopen.bmj.com).

If you have any questions on BMJ Open's open peer review process please email info.bmjopen@bmj.com

BMJ Open

Effect of Nepal Perinatal Quality Improvement Project on patient satisfaction: a cluster randomized control trial

Journal:	*BMJ Open*
Manuscript ID	bmjopen-2021-054544
Article Type:	Original research
Date Submitted by the Author:	12-Oct-2021
Complete List of Authors:	Brunell, Olivia; Uppsala University, Department of Women's and Children's Health<br>Chaulagain, Dipak; Uppsala University, Department of Women's and Children's Health<br>KC, Ashish; Uppsala University, Department of Women's and Children's Health<br>Bergström, Anna; Uppsala University, Department of Women's and Children's Health<br>Målqvist, Mats; Uppsala University, Department of Women's and Children's Health
Keywords:	PAEDIATRICS, NEONATOLOGY, Quality in health care < HEALTH SERVICES ADMINISTRATION & MANAGEMENT

SCHOLARONE™
Manuscripts

I, the Submitting Author has the right to grant and does grant on behalf of all authors of the Work (as defined in the below author licence), an exclusive licence and/or a non-exclusive licence for contributions from authors who are: i) UK Crown employees; ii) where BMJ has agreed a CC-BY licence shall apply, and/or iii) in accordance with the terms applicable for US Federal Government officers or employees acting as part of their official duties; on a worldwide, perpetual, irrevocable, royalty-free basis to BMJ Publishing Group Ltd ("BMJ") its licensees and where the relevant Journal is co-owned by BMJ to the co-owners of the Journal, to publish the Work in this journal and any other BMJ products and to exploit all rights, as set out in our licence.

The Submitting Author accepts and understands that any supply made under these terms is made by BMJ to the Submitting Author unless you are acting as an employee on behalf of your employer or a postgraduate student of an affiliated institution which is paying any applicable article publishing charge ("APC") for Open Access articles. Where the Submitting Author wishes to make the Work available on an Open Access basis (and intends to pay the relevant APC), the terms of reuse of such Open Access shall be governed by a Creative Commons licence – details of these licences and which Creative Commons licence will apply to this Work are set out in our licence referred to above.

Other than as permitted in any relevant BMJ Author's Self Archiving Policies, I confirm this Work has not been accepted for publication elsewhere, is not being considered for publication elsewhere and does not duplicate material already published. I confirm all authors consent to publication of this Work and authorise the granting of this licence.

For peer review only

Effect of Nepal Perinatal Quality Improvement Project on patient satisfaction: a cluster randomized control trial

Olivia Brunell[1], Dipak Chaulagain[1], Ashish KC[1], Anna Bergström[1], Mats Målqvist[1]

[1] Uppsala Global Health Research on Implementation and Sustainability (UGHRIS), Department of Women's and Children's Health, Uppsala University

Corresponding author

Olivia Brunell, Department of Women's and Children's Health, Uppsala University, Sjukhusvägen 7, 753 09 Uppsala, Sweden

e-mail: olivia.brunell@kbh.uu.se
phone: +46 8611 00 00

Keywords

Neonatology, Perinatal care, Quality Improvement, Patient Satisfaction

Word count

3573

For peer review only

ABSTRACT

Introduction To end preventable neonatal deaths, good quality of perinatal care in health facilities is crucial. Patient's experience of care is an important aspect of quality of care and can be measured by the patient-centered outcome patient satisfaction.

Objective To investigate the effect of a quality improvement (QI) intervention on patients' satisfaction.

Design Stepped-wedge cluster randomized controlled design. Participating hospitals were randomized by size into four different wedges.

Setting 12 secondary-level public hospitals in Nepal.

Participants Women who gave birth in the hospitals at a gestational age of ≥22weeks, with fetal heart sound at admission. Adverse outcomes were excluded. One hospital was excluded due to data incompleteness and four low-volume hospitals due to large heterogeneity. The final analysis included 54,919 women.

Intervention Hospital management was engaged and facilitators were recruited from within the hospitals. Available perinatal care was assessed in each hospital, followed by a bottle-neck analysis workshop. A three-day training in essential newborn care was carried out for medical staff, and a set of quality improvement tools were introduced to be used in every day practice (including skill-checks, self- assessment checklists, scoreboards, and bi-weekly Plan-Do-Study-Act-meetings). A refresher training was held after 6 months.

Outcome measures Women's experience and satisfaction with care at discharge.

Results The likelihood of women being satisfied with care increased after the intervention, (adjusted odds ratio [aOR] 1.66, 95% CI: 1.59-1.73) but the results varied between hospitals (aOR 0,39-3,57). The proportions of satisfaction were low (control 58%, intervention 62%).

Conclusions This QI-intervention on essential newborn care improved patient satisfaction and has potential to improve both clinical practices and patient-centered outcomes. We recommend further studies on which aspects of care that are of most importance to improve women's satisfaction with perinatal care in hospitals in Nepal.

Trial registration ISRCTN30829654

For peer review only

ARTICLE SUMMARY

Strengths and limitations of this study

- This study focuses on patients' experience of care, an important dimension of quality improvement which is often overlooked when evaluating quality improvement efforts
- The large sample size from different hospitals increases representativeness of the sample and generalizability of our results, while external validity is strengthened by the use of the WHO framework
- The stepped-wedge cluster-randomization allowed us to eventually roll out the intervention at all sites, while mimicking a randomized-controlled trial
- The exclusion of one high-volume hospital and the four low- volume hospitals, deviates from the original stepped-wedged design and should be considered when interpreting the results
- We measured the WHO domains of experience of care and no other factors that are also known to affect satisfaction, such as waiting times, overcrowding and physical resources in the health facility

For peer review only

INTRODUCTION

In 2017 an estimated 2,5 million children died in the neonatal period whereof about 1 million newborn died during their first day of life[1]. Though an increasing number of women are giving birth in health facilities it has not ensured improved health care outcomes[2]. Improving the quality of perinatal care provided in health facilities is crucial to end preventable mortality[2-4]. Both the definition of Quality of Care (QoC) and approaches to its assessment are subjects for discussion and there are no universal consensus on these matters[5]. The definition of QoC by the World Health Organization (WHO) is "The extent to which health care services provided to individuals and patient populations improve desired health outcomes" and to achieve this, health care must be safe, effective, timely, efficient, equitable and people-centered. People-centered care means providing care to individuals considering their preferences, needs and values and the culture of their communities[6]. It is important both because patients have the right to be treated with dignity and respect but also because it is associated with improved health care utilization and health outcomes[7]. In 2016, WHO developed a framework and standards with accompanying quality measures for the quality of maternal and newborn health care. In this framework, QoC is divided into two interlinked dimensions; (1) *Provision of clinical care* by health workers, and (2) patients' *Experience of care*[8] (Figure 1). *Experience of care* is a people-centered measure, a process indicator which reflects the interactions that patients have with the healthcare system[9]. It is composed of three major domains; effective communication, respect and dignity, and emotional support[6]. Outcomes of *Experience of care* in the framework are people-centered outcomes, such as *Patient satisfaction,* which reflects whether or not the care has met the patients' needs and expectations[9-11].

In 2013 the research group conducted a study of the implementation of a quality improvement (QI) package for neonatal resuscitation in a tertiary hospital in Nepal. In this study the Helping Babies Breathe (HBB) program, developed by The American Academy of Pediatrics to improve health workers' performance on basic resuscitation [12], was implemented. In addition to HBB-training, the intervention included QI-components, aiming to continuously reinforce the HBB protocol throughout the intervention period and to motivate both hospital management and individual health workers to be engaged in improvement.

The study, Helping Babies Breath-Quality Improvement Cycle (HBB-QIC), showed improvement in health workers adherence to neonatal resuscitation protocols and a large

For peer review only - http://bmjopen.bmj.com/site/about/guidelines.xhtml

decrease in intrapartum still births and first-day neonatal mortality [13]. These positive results are more pronounced than in previous studies of implementation of the HBB-program [14, 15] and can be due to the addition of the QI components.

However, there were no significant change of overall in-hospital perinatal mortality, which indicates that efforts are also needed to improve the continued postnatal care[16]. Based on the findings from HBB-QIC, a scale-up was developed by the research group in collaboration with Ministry of Health and Population in Nepal. This is called Nepal Perinatal Quality Improvement Project (NePeriQIP) and aims to improve quality of perinatal care through increased clinical skills and knowledge in essential newborn care (ENC) and establish a structure for continuous quality improvement[17]. The primary objective of the NePeriQIP trial was to evaluate impact on intrapartum mortality (intrapartum stillbirth and first-day mortality), and the results showed a significant reduction in intrapartum-related deaths (intrapartum stillbirths and first day mortality) in the intervention period (aOR, 0.79; 95% CI, 0.69–0.92)[18]. Secondary outcomes are now under investigation and this current study aims to evaluate the effect of NePeriQIP on *patient satisfaction*.

METHODS

Trial design

A stepped-wedge cluster-randomized design was used[17], where 12 hospitals were randomized by size into four wedges. Each wedge included one high-volume (>8000 deliveries a year), one medium-volume (>3000 deliveries a year) and one low-volume (>1000 deliveries a year) hospital. Each cluster had a control and an intervention period. During the first three months of the study period no intervention activities took place at any of the hospitals (baseline period). NePeriQIP was then introduced to one wedge with three hospitals at a time, with a three months delay between wedges, and eventually all hospitals were exposed to the intervention (Figure 2).

Setting

The selected 12 hospitals filled the criteria of having >1000 deliveries per year and being a referral center for maternal and newborn care. Although most of the hospitals were located in the flatlands, they were diverse in relation to ethnicity, language and religion. All hospitals provided vaginal and cesarian deliveries, and had access to neonatal resuscitation services at birth. Skilled birth-attendants led the labor units. Medical doctors led the pediatric units where

For peer review only - http://bmjopen.bmj.com/site/about/guidelines.xhtml

sick newborns were managed in the low-volume hospitals. Pediatricians led the specialized sick newborn care units in the medium and high-volume hospitals. The intrapartum related mortality rate (intrapartum stillbirth and first day neonatal mortality) ranged from 9 to 31 per 1,000 births at the hospitals (mean 13/1000 births), during the baseline period[18].

Participants

All pregnant women who gave birth at any of the 12 hospitals after the baseline period, who gave their oral and written consent, who were at gestational age of 22 weeks or more and who had fetal heart sound at admission were eligible for inclusion in the study. Women that experienced stillbirths, neonatal deaths, malformations and complications during delivery were excluded as these events could bias patient satisfaction.

The intervention

The development of the intervention was guided by the integrated-Promoting Action on Research Implementation in Health Services (iPARIHS) framework [19]. Ministry of Health, together with the research team, developed an implementation guideline for the implementation process. In each hospital, the QI-package was introduced in the following steps: First, the hospital management was oriented on the QI-package. They appointed in-hospital facilitators from among pediatricians, medical officers and nurses to facilitate the implementation, supported by the study team. The number of facilitators depended on the size of the hospital: two from low-volume, three from medium-volume and four from high-volume-hospitals. The facilitators participated in a seven-day training on the QI-package, provided by the study team. Following the training, the QI facilitators assessed service readiness and availability in their respective hospital, using a checklist developed by the team, and a bottleneck analysis workshop was organized based on that. After these initial steps, a three-day on-site training for all health staff involved in perinatal care was organized by the facilitators and the study team. The training consisted of education and skills-training in ENC and implementation of a set of QI-tools. The ENC component included HBB and immediate newborn care (thorough drying, skin-to-skin contact, delayed clamping of the cord and early initiation of breastfeeding) and were taught using the HBB training manual version one[20]. In addition, lectures were given on breastfeeding, infection prevention and kangaroo mother care for low birth-wight newborns. The QI- tools included daily bag-and-mask ventilation skill check on an HBB-mannequin, checklist for preparations before every delivery, self-assessment checklist of performed resuscitation, and use of score-boards comprising major

For peer review only - http://bmjopen.bmj.com/site/about/guidelines.xhtml

indicators on neonatal resuscitation. In addition to these tools a structured model for quality improvement, Plan-Do-Study-Act (PDSA)-cycle , was used aiming to identify and act upon local problems [21]. A one-day refresher training in HBB was held for health staff after six months.

Conceptual framework

The WHO conceptual framework on QoC (Figure 1)[6] and the WHO-document "Standards for improving quality of maternal and newborn care in health facilities"[8] were used to guide data collection and analyzes for this study. In the framework, QoC consists of the two inter-linked dimensions Provision of care and Experience of care. Experience of care contains three domains; *effective communication*, *preservation of respect and dignity*, and *emotional support*. Though NePeriQIP focus on clinical provision of care, a successful implementation where health care workers are more engaged in the quality of perinatal care could generate better experiences among patients and manifest in patient satisfaction.

Data collection and variables

The study period followed the Nepali calendar and was initiated on June 2017 and ran for 15 months up until October 2018. Data was collected through structured interviews with all included mothers upon discharge, throughout the study period. An independent data collection team was established at each hospital and a structured questionnaire developed by the study team and piloted on beforehand, was used to collect the data. After completion of the data collection forms, they were sent weekly to the central research office in Kathmandu, where they were entered into a Census and Survey Processing System database by a team of independent data entry officers.

The **primary outcome** was *Patient satisfaction*. This was measured as an aggregate score of two questions;

(1) *Overall, how satisfied are you with the services?*
   (Recorded on a Likert scale of 1 to 5. 1= very dissatisfied, 2= dissatisfied, 3= neither, 4= satisfied, 5= very satisfied. Scores were dichotomized, with an answer of 4 or more being set as satisfied).

(2) *Would you recommend a friend to deliver at this hospital?*
   (recorded yes/ no)

For peer review only

To be considered satisfied with services received, mothers needed to respond favorably to both questions.

The **secondary outcome** was the three domains of *Experience of care* (effective communication, respect and dignity, and emotional support) in the WHO Quality of Care framework for maternal and newborn health [6]. It was investigated through a set of questions suggested as quality measures of experience of care in the WHO-document "Standards for improving quality of maternal and newborn care in health facilities"[8].

(1) Patient communication

Were you adequately informed by the care provider about examinations, actions and decisions taken for your care throughout the hospital stay?

(recorded yes/no)

Are you satisfied with health education and information received from health care providers?

(Recorded on a Likert scale of 1 to 5. 1= very dissatisfied, 2= dissatisfied, 3= neither, 4= satisfied, 5= very satisfied. Scores were dichotomized, with an answer of 4 or more being set as satisfied).

(2) Respect and dignity

Are you satisfied with the degree of privacy during your stay in labor and child birth areas?

(Recorded on a Likert scale of 1 to 5. 1= very dissatisfied, 2= dissatisfied, 3= neither, 4= satisfied, 5= very satisfied. Scores were dichotomized, with an answer of 4 or more being set as satisfied).

Where you or your newborn physically, verbally or sexually abused during labor or childbirth or after birth?

(recorded yes/no)

Were you treated with respect and was your dignity preserved during your stay at the hospital?

(recorded yes/no)

(3) Emotional support

Did you have a companion of your choice during labor and childbirth?

For peer review only - http://bmjopen.bmj.com/site/about/guidelines.xhtml

(recorded yes/no)

Statistical methods

There was no a priori estimation of sample size for this sub-study, it was calculated for the primary outcome of the larger NePeriQIP-trial, intrapartum mortality[18] . Pearson's Chi-Squared test was used to analyze background characteristics of participants in the control and intervention groups, performed with Statistical Package for Social Sciences (SPSS) version 25.0. Change in satisfaction and experiences of care between the control and intervention groups was analyzed by generalized linear mixed models (GLMM) using the software R (version 3.4.0). Analyses were performed with R package lme4. Adjustments were made for structural factors (cast/ethnicity and educational level). Intra-cluster correlations coefficients (ICC) for each outcome were calculated. Initial analyses showed high ICC for multiple variables, making the results inconclusive. Further analysis of the data by hospital displayed that the low-volume hospitals showed large heterogeneity. As a consequence, the final GLMM analysis was performed on the medium and high-volume hospitals. Analysis of change before and after intervention at each hospital was performed with binary logistic regression of satisfaction and experience of care (adjusted for caste/ethnicity and education level) with Statistical Package for Social Sciences (SPSS) version 25.0.

Ethical considerations

Ethical approval was obtained from the Nepal Health Research Council (ref 26–2017). The data used in this study was collected through face-to-face interviews after obtaining written informed consent from the participants. The identity of the participants was coded after data collection, and the code key was stored in a safe environment that only the project management had access to.

Patient and public involvement

Patients were not directly involved in developing this study, however it was planned and conducted in collaboration with Ministry of Health in Nepal.

For peer review only - http://bmjopen.bmj.com/site/about/guidelines.xhtml

RESULTS

A total number of 65,895 women were eligible for inclusion in this study, whereof 946 women were excluded based on exclusion criteria. Due to data incompleteness from one of the hospitals, another 5,366 women were excluded, leaving 59,583 women available for analysis. Initial analysis revealed that low-volume hospitals showed large heterogeneity and differed from the medium and high-volume hospitals. As a consequence of this finding, the final GLMM analysis was performed on the medium and high-volume hospitals where 92% of deliveries took place, n= 54,919 (Figure 3).

Characteristics

A majority of the women had an education higher than primary (69%) and belonged to an advantageous cast (67%). About half of the women were first-time mothers (51%) but few were adolescents (7.6%) and the coverage of antenatal care was high (80% had four antenatal care visits before delivery). The cesarean section rate was 19% and most of them (73%) were emergency cesarean sections. The proportion of preterm deliveries were 13% and babies with low birth weight were 14% (Table 1).

Outcomes

Women were more likely to be satisfied with care after the intervention than before, adjusted Odds Ratio (aOR) 1.66 [CI: 1.59-1.73, ICC: 0.275]). The results varied between hospitals (aOR 0.39-3.57) (Table S1) and the overall proportion of satisfaction was low. Prior to the intervention a proportion of 58% of the women were satisfied, which increased to 62% after (Table 2).

The domains of experience of care were affected in a positive direction by the intervention. Women were more likely to be *satisfied with education and information* from health workers after implementation (aOR 1.34 [CI: 1.29-1.40], ICC: 0.167) as well as more likely to state that they had been *treated with preserved dignity and respect* (aOR: 1.81 [CI: 1.52-2.16], ICC: 0.063]). The likelihood of *Experience abuse during the hospital stay* decreased after the implementation (aOR: 0.42 [CI: 0.34-.051, ICC: 0.082]) and the likelihood of being *satisfied with the level of privacy* increased (aOR 1.14 [CI: 1.09-1.18, ICC: 0.136]) (Table 2). Two measures seemed to have been affected in a negative direction after the intervention: the likelihood of being *adequately informed about examinations, actions and decisions taken for their care* (aOR 0.55 [CI: 0.53-0.58, ICC: 0.507] and *having a companion of choice during*

For peer review only - http://bmjopen.bmj.com/site/about/guidelines.xhtml
For peer review only

labor (aOR 0.27 [CI:0.25-0.29, ICC: 0.687]. These two measures, however, displayed relatively high ICC in the GLMM model, rendering the adjusted odds ratios to be inconclusive. (Table 2).

A very high proportion of women stated that they were treated with dignity and respect (before intervention 98.7%, after 99.1%) and very few had experienced abuse during their hospital stay (1.1% before intervention and 0.6% after). The proportion of women who stated that they were adequately informed about their care were 76.3% before intervention and 80.6% after. However, not many women had a companion of choice during labor (16.8% before and 19.0% after), and the proportions of women who were satisfied with the information and education from health workers, and with the level of privacy, were also low (43.3% before and 49.3% after, 51.6% before and 51.0% after, respectively) (Table 2).

For peer

DISCUSSION

The quality of perinatal care in health facilities needs to be improved to save newborn lives [2-4]. Measuring patient satisfaction is important in several aspects to improve quality of care; It can reveal gaps in the health system to address, it creates accountability among health care providers and stakeholders, it is useful to guide and evaluate QI efforts, and in addition satisfaction is known to affects patients care-seeking behavior and compliance[9, 22-24]. Our study shows that after the implementation of the QI-intervention NePeriQIP, the likelihood that women were satisfied with perinatal care increased. Satisfaction is an outcome measure of experience of care [9], and in our study the people-centered quality measures of experience of care suggested by WHO were affected in a positive direction, along with satisfaction. The exact mechanisms behind our results are not clear as the intervention did not specifically focus on people-centered skills. However, besides that NePeriQIP focused on improvement of provision of care through training, it also included QI-tools to strengthen professional attitudes among health workers and sustain change in practice. Self-evaluation, insight in the ongoing progress and involvement in problem-solving promote a higher level of learning which can create a deeper understanding among health workers and make them pay more attention to what they are doing and why [25]. This can improve their capacity to inform and educate patients, to handle stressful situations and provide more time and engagement to attend to interpersonal interactions with patients. With increased knowledge and continuous

For peer review only - http://bmjopen.bmj.com/site/about/guidelines.xhtml

discussions concerning care of the mothers and newborn, healthcare workers might also get more sensitized and empathic towards their patients.

However, satisfaction is a complex phenomenon influenced by several determinants, and we cannot conclude that the improvement in satisfaction is explained solely by improvement in the measures of experience of care that we have studied. Other known determinants of satisfaction are structural elements (i.e., cleanliness of the health facility and availability of human recourses, medicines and supplies) and outcome related determinants such as health status of the mother and newborn[26]. Nevertheless, it is previously shown that the inter-personal components of experience of care dominate satisfaction with maternity care in Nepal and other low-income countries[26, 27].

Though we could see an increased satisfaction after the intervention, the proportion of satisfied women both before and after was low. This despite that women tend to report more satisfied with care if interviewed early after childbirth, biased by social desirability and the joy of having a healthy baby [28] and previous studies of satisfaction in Nepal has shown higher levels [29, 30]. We do not know why the women were not more satisfied with care but some factors can be considered. First, we sought to reduce the influence of social desirability by our definition of satisfaction (overall satisfaction combined with whether to recommend the facility to a friend). Other aspects that could play a role are the low proportions of women who were satisfied with education and information from health workers and with the privacy, and the fact that many women did not have a companion of choice during labor. In addition, other determinants that was not the scope of our study, could be of importance. Other studies of satisfaction with perinatal care in Nepal has shown that having a protected waiting area and having an opportunity to ask questions are positively associated with patient satisfaction, while over-crowding and long waiting times has been shown to increase the likelihood of dissatisfaction[27, 30].

We adjusted for the structural determinants cast/ethnicity and educational level, which can be grounds for discrimination, altered treatment and outcomes. Many characteristics differed between control and intervention period (Table 1), but most of them were considered intermediate determinants and were thus omitted from the analysis. Given the complexity of the stepped-wedge design, no further multi-level analyses were performed.

For peer review only - http://bmjopen.bmj.com/site/about/guidelines.xhtml

The main strengths of our study are the large sample and the stepped-wedge cluster-randomization. The latter allowed us to eventually roll out the intervention at all sites, while mimicking a randomized-controlled trial. Our study was also guided by WHO framework which strengthens external validity.

There are some limitations to our study worth mentioning. We excluded women with adverse outcomes since this could over shadow the experience of care and skew our results. We had to exclude one high-volume hospital due to data incompleteness and the four low-volume hospitals due to initial analysis showing high ICC. This deviates from the original stepped-wedged design and should be considered when interpreting the results. In this study we only focused on measuring the WHO domains of experience of care and no other factors that are also known to affect satisfaction, such as waiting times, overcrowding, patients' expectations, provision of medical care or physical resources in the health facility[27, 31]. A more in-depth understanding of the women's satisfaction could have been achieved by adding qualitative interviews.

CONCLUSION

We found that after the implementation of NePeriQIP, women giving birth in health facilities were significantly more likely to be satisfied with care. This indicates that this QI-intervention has the potential to improve both dimensions of quality of care highlighted by WHO to improve health outcomes, namely Provision of care and Experience of care. Our results underline the interaction between the dimensions, though the exact mechanisms are still to be investigated. As satisfaction with perinatal care in Nepal still needs improvement, we recommend further studies on these mechanisms, and on which aspects of care that are of most importance to improve patient satisfaction.

ACKNOWLEDGEMENTS

The authors would like to thank the management and health care staff of all participating hospitals as well as facilitators, mentors, data collectors, and the data management team at Golden Community. Thanks to Abhishek Gurung, Elisha Joshi, Sunil Gajurel, Prajwal Poudel and Asmita Paudel for facilitating the QI implementation process in hospitals. Special thanks to Omkar Basnet for excellent work in data management and to Regina Gurung for

For peer review only

participating in managing the project. Lastly, we would like to express our gratitude towards all participating mothers.

ADDITIONAL INFORMATION

Author Contributions OB, MM, AB and AK conceptualized the study. AK and DC supervised data collection and management. OB and MM analyzed data and prepared the first draft. All authors, reviewed, commented and approved the final version of the manuscript.

Trial registration number ISRCTN30829654

The full trial protocol "Scaling up quality improvement intervention for perinatal care in Nepal (NePeriQIP); study protocol of a cluster randomized trial" can be accessed at BMJ Global Health[17]

Funding This work was supported by Swedish Research Council (2016-05621), the Laerdal Foundation for Acute Medicine, Norway (40198), and Einhorn Family Foundation, Sweden. The funders had no role in study design, data collection and analysis, decision to publish, or preparation of the manuscript.

Competing interests There are no competing interests for any author.

For peer review only

REFERENCES

1. Hug L, Sharrow D, Zhong K, You D: **Levels & Trends in Child Mortality**. In.: United Nations Inter-agency Group for Child Mortality Estimation (UN IGME); 2018.
2. Scott KW, Jha AK: **Putting quality on the global health agenda**. *N Engl J Med* 2014, **371**(1):3-5.
3. Kruk M, Gage A, Joseph N, Danaei G, Garcia-Saisó S, Salomon JA: **Mortality due to low-quality health systems in the universal health coverage era: a systematic analysis of amenable deaths in 137 countries**. *The Lancet* 2018, **392**(10160):2203-2212.
4. Bhutta Z, Das JK, Bahl R, Lawn JE, Salam RA, Vinod K Paul VK, Sankar MJ, Blencowe H, Rizvi A, Chou VB *et al*: **Every Newborn: Can available interventions end preventable deaths in mothers, newborn babies, and stillbirths, and at what cost?** . *The Lancet* 2014.
5. Donabedian A: **Evaluating the quality of medical care. 1966**. *Milbank Q* 2005, **83**(4):691-729.
6. Tuncalp, Were WM, MacLennan C, Oladapo OT, Gulmezoglu AM, Bahl R, Daelmans B, Mathai M, Say L, Kristensen F *et al*: **Quality of care for pregnant women and newborns-the WHO vision**. *BJOG* 2015, **122**(8):1045-1049.
7. Doyle C, Lennox L, Bell D: **A systematic review of evidence on the links between patient experience and clinical safety and effectiveness**. *BMJ Open* 2013, **3**(1).
8. WHO.: **Standards for improving quality of maternal and newborn care in health facilities**. In. Geneva: WHO Press; 2016.
9. Larson E, Sharma J, Bohren MA, Tuncalp O: **When the patient is the expert: measuring patient experience and satisfaction with care**. *Bull World Health Organ* 2019, **97**(8):563-569.
10. Donabedian A: **The Definition of Quality and Approaches to Its Assessment, vol 1: Explorations in Quality Assessment and Monitoring**. University of Michigan: Health Administration Press; 1980.
11. Cleary PD, McNeil BJ: **Patient satisfaction as an indicator of quality care**. *Inquiry* 1988, **25**(1):25-36.
12. Wall SN, Lee AC, Carlo W, Goldenberg R, Niermeyer S, Darmstadt GL, Keenan W, Bhutta ZA, Perlman J, Lawn JE: **Reducing intrapartum-related neonatal deaths in low- and middle-income countries-what works?** *Semin Perinatol* 2010, **34**(6):395-407.
13. Kc A, Wrammert J, Clark RB, Ewald U, Vitrakoti R, Chaudhary P, Pun A, Raaijmakers H, Malqvist M: **Reducing Perinatal Mortality in Nepal Using Helping Babies Breathe**. *Pediatrics* 2016, **137**(6).
14. Msemo G, Massawe A, Mmbando D, Rusibamayila N, Manji K, Kidanto HL, Mwizamuholya D, Ringia P, Ersdal HL, Perlman J: **Newborn mortality and fresh stillbirth rates in Tanzania after helping babies breathe training**. *Pediatrics* 2013, **131**(2):e353-360.
15. Goudar SS, Somannavar MS, Clark R, Lockyer JM, Revankar AP, Fidler HM, Sloan NL, Niermeyer S, Keenan WJ, Singhal N: **Stillbirth and newborn mortality in India after helping babies breathe training**. *Pediatrics* 2013, **131**(2):e344-352.

For peer review only - http://bmjopen.bmj.com/site/about/guidelines.xhtml

16. Wrammert J, Kc A, Ewald U, Malqvist M: **Improved postnatal care is needed to maintain gains in neonatal survival after the implementation of the Helping Babies Breathe initiative**. *Acta Paediatr* 2017, **106**(8):1280-1285.
17. Kc A, Bergstrom A, Chaulagain D, Brunell O, Ewald U, Gurung A, Eriksson L, Litorp H, Wrammert J, Gronqvist E *et al*: **Scaling up quality improvement intervention for perinatal care in Nepal (NePeriQIP); study protocol of a cluster randomised trial**. *BMJ Glob Health* 2017, **2**(3):e000497.
18. Kc A, Ewald U, Basnet O, Gurung A, Pyakuryal SN, Jha BK, Bergstrom A, Eriksson L, Paudel P, Karki S *et al*: **Effect of a scaled-up neonatal resuscitation quality improvement package on intrapartum-related mortality in Nepal: A stepped-wedge cluster randomized controlled trial**. *PLoS Med* 2019, **16**(9):e1002900.
19. Harvey G, Kitson A: **PARIHS revisited: from heuristic to integrated framework for the successful implementation of knowledge into practice**. *Implement Sci* 2016, **11**:33.
20. Singhal N, Lockyer J, Fidler H, Keenan W, Little G, Bucher S, Qadir M, Niermeyer S: **Helping Babies Breathe: global neonatal resuscitation program development and formative educational evaluation**. *Resuscitation* 2012, **83**(1):90-96.
21. Christoff P: **Running PDSA cycles**. *Curr Probl Pediatr Adolesc Health Care* 2018, **48**(8):198-201.
22. Al-Abri R, Al-Balushi A: **Patient satisfaction survey as a tool towards quality improvement**. *Oman Med J* 2014, **29**(1):3-7.
23. Quintana JM, Gonzalez N, Bilbao A, Aizpuru F, Escobar A, Esteban C, San-Sebastian JA, de-la-Sierra E, Thompson A: **Predictors of patient satisfaction with hospital health care**. *BMC Health Serv Res* 2006, **6**:102.
24. Bernhart MH, Wiadnyana IG, Wihardjo H, Pohan I: **Patient satisfaction in developing countries**. *Soc Sci Med* 1999, **48**(8):989-996.
25. Anderson L.W. KDR, Bloom B.S: **A Taxonomy for Learning, Teaching, and Assessing : a Revision of Bloom's Taxonomy of Educational Objectives / Editors, Lorin W. Anderson, David Krathwohl ; Contributors, Peter W. Airasian ... [et Al.]**. . New York: Longman; 2001.
26. Srivastava A, Avan BI, Rajbangshi P, Bhattacharyya S: **Determinants of women's satisfaction with maternal health care: a review of literature from developing countries**. *BMC Pregnancy Childbirth* 2015, **15**:97.
27. Mehata S, Paudel YR, Dariang M, Aryal KK, Paudel S, Mehta R, King S, Barnett S: **Factors determining satisfaction among facility-based maternity clients in Nepal**. *BMC Pregnancy Childbirth* 2017, **17**(1):319.
28. Lumley J: **Assessing satisfaction with childbirth**. *Birth* 1985, **12**(3):141-145.
29. Ministry of Health and Population (MOHP) [Nepal] HRaSDFH, and Nepal Health Sector Support Programme (NHSSP): **Service Tracking Survey 2013**. In. Kathmandu, Nepal: Ministry of Health and Population, HERD, and NHSSP, Ramsah Path, Kathmandu, Nepal; 2014.
30. Acharya S, S. Sharma, B. Dulal, K. Aryal: **Quality of Care and Client Satisfaction with Maternal Health Services in Nepal: Further Analysis of the 2015 Nepal Health Facility Survey. DHS Further Analysis Reports No. 112**. In. Rockville, Maryland, USA: ICF; 2018.
31. Bjertnaes OA, Sjetne IS, Iversen HH: **Overall patient satisfaction with hospitals: effects of patient-reported experiences and fulfilment of expectations**. *BMJ Qual Saf* 2012, **21**(1):39-46.

For peer review only

TABLES

Table 1. Characteristics of study participants in control and intervention group. Chi2-test to detect group differences before and after NePeriQIP intervention start (n_{tot}=59,583)

Demographic characteristics		Total n (%)	Control n (%)	Intervention n (%)	p-value
Adolescent mother (≤19 years)	No	55,061 (92.4)	22,026 (92.5)	33,035 (92.3)	0.415
	Yes	4,522 (7.6)	1,781 (7.5)	2,741 (7.7)	
Elder mother (>35 years)	No	59,012 (98.9)	23,667 (98.9)	35,345 (98.8)	0.082
	Yes	681 (1.1)	250 (1.1)	431 (1.2)	
Mother's cast/ethnicity	relatively disadvantaged	19,667 (33.0)	8,537 (35.9)	11,130 (31.1)	<0.001*
	relatively advantaged	39,916 (67.0)	15,270 (64.1)	24,646 (68.9)	
Mother's education	Primary or less	18,591 (31.3)	7,572 (31.9)	11,019 (30.9)	0.006*
	Secondary or higher	40,829 (68.7)	16,139 (68.1)	24,690 (69.1)	
**Pregnancy characteristics**					
Parity	Nulli	30,610 (51.4)	12,400 (52.1)	18,210 (50.9)	0.005*
	Multi	28,973 (48.6)	11,407 (47.9)	17,566 (49.1)	
>Four ANC visits	No	11,700 (19.9)	5,136 (21.9)	6,564 (18.5)	0.000*
	Yes	47,131 (80.1)	18,273 (78.1)	28,858 (81.5)	
**Delivery characteristics**					
Spontaneous vaginal	No	14,628 (23.3)	5,294 (22.2)	8,704 (24.3)	<0.001*
	Yes	45,585 (76.7)	18,513 (77.8)	27,072 (75.7)	
Elective CS	No	56,563 (94.9)	22,413 (94.1)	34,150 (95.5)	<0.001*
	Yes	3,020 (5.1)	1,394 (5.9)	1,626 (4.5)	
Emergency CS	No	51,559 (86.5)	20,846 (87.6)	30,713 (85.8)	<0.001*
	Yes	8,024 (13.5)	2,961 (12.4)	5,063 (14.2)	
**Newborn characteristics**					
Preterm	No	51,579 (86.6)	21,740 (91.3)	29,839 (83.4)	<0.001*
	Yes	8,004 (13.4)	2,067 (8.7)	5,937 (16.6)	
LBW	No	46,997 (86.2)	20,540 (87.4)	26,457 (85.3)	<0.001*
	Yes	7,524 (13.8)	2,949 (12.6)	4,575 (14.7)	
Sex of baby	Boy	32,132 (54.1)	12,771 (53.9)	19,361 (54.2)	0.391
	Girl	27,288 (45.9)	10,940 (46.1)	16,348 (45.8)	

For peer review only - http://bmjopen.bmj.com/site/about/guidelines.xhtml

Table 2. Binary logistic regression for full sample and GLMM analysis correcting for clustering (by hospital) displaying adjusted odds ratios (n=54,919).

				Generalized Linear Mixed Method (GLMM)	
		Control N(%)	Intervention N(%)	aOR (95% CI)**	Intra-class correlation (ICC)
**Patient satisfaction**					
Are you satisfied with services and would you recommend a friend?	No	9070 (42.1)	12694 (38.0)	Ref	
	Yes	12485 (57.9)	20670 (62.0)	1.66 (1.59-1.73)	0.275
**Patient experience**					
Were you adequately informed by the care provider about examinations, actions and decisions taken for your care?	No	5116 (23.7)	6482 (19.4)	Ref	
	Yes	16439 (76.3)	26882 (80.6)	0.55 (0.53-0.58)	0.507
Are you satisfied with education/information?	No	12227 (56.7)	16922 (50.7)	Ref	
	Yes	9328 (43.3)	16422 (49.3)	1.34 (1.29-1.40)	0.167
Are you satisfied with level of privacy?	No	10433 (48.4)	16350 (49.0)	Ref	
	Yes	11122 (51.6)	17014 (51.0)	1.14 (1.09-1.18)	0.136
Were you or your newborn physically, verbally or sexually abused during labor or childbirth or after birth?	No	21308 (98.9)	33149 (99.4)	Ref	
	Yes	247 (1.1)	215 (0.6)	0.42 (0.34-0.51)	0.082

For peer review only - http://bmjopen.bmj.com/site/about/guidelines.xhtml

Were you treated with respect and was your dignity preserved during your stay at the hospital?	No	275 (1.3)	290 (0.9)	Ref	
	Yes	21280 (98.7)	33074 (99.1)	1.81 (1.52-2.16)	0.063
Did you have a companion of your choice during labor and child birth?	No	17932 (83.2)	27036 (81.0)	Ref	
	Yes	3623 (16.8)	6328 (19.0)	0.27 (0.25-0.29)	0.687

*Binary logistic regression models adjusted for caste/ethnicity and education level

** GLMM models adjusted for clustering (by hospital) and caste/ethnicity and education level

For peer review only

Figure legends

Figure 1. WHO Framework for the Quality of maternal and newborn health care. Reprinted from the WHO document "Standards for improving quality of maternal and newborn care in health facilities", Page No. 16, Copyright World Health Organization (2016).

Figure 2. Stepped-wedge design and time line of the NePeriQIP trial. The period within bold lines represents the baseline period. The shaded period constitutes the intervention period. Each wedge contains three hospitals (clusters).

Figure 3. Participants flow diagram

For peer review only

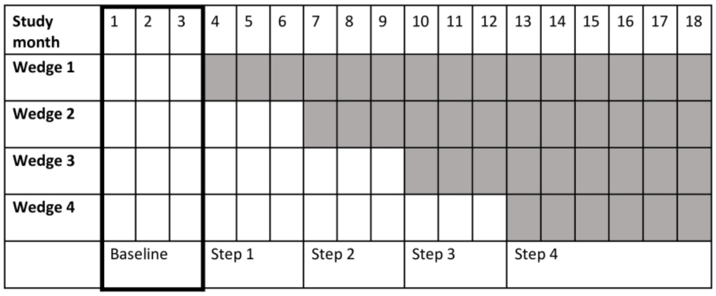

Figure 2. Stepped-wedge design and time line of the NePeriQIP trial. The period within bold lines represents the baseline period. The shaded period constitutes the intervention period. Each wedge contains three hospitals (clusters).

209x297mm (300 x 300 DPI)

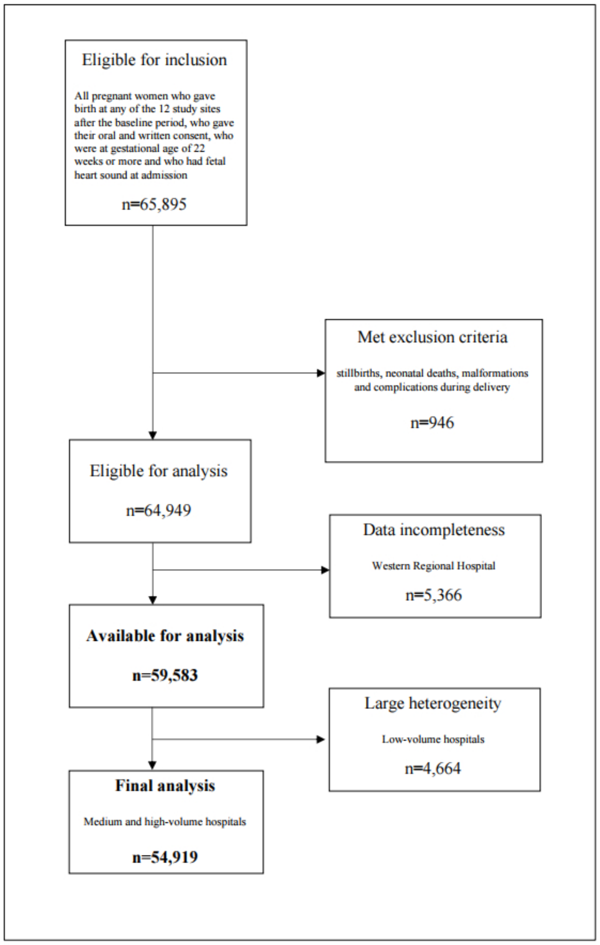

Figure 3. Participants flow diagram

209x296mm (96 x 96 DPI)

Table S1. Binary logistic regression of patient satisfaction and quality measures of experience of care, by hospital. Adjusted for caste/ethnicity and education level (n=54,919).

		**Satisfaction**			**Informed**			**Educated**			**Privacy**			**Abuse**			**Respect**			**Companion**		
**Hospital**		Yes N(%)	No N(%)	Adj OR (95% CI)	Yes N(%)	No N(%)	Adj OR (95% CI)	Yes N(%)	No N(%)	Adj OR (95% CI)	Yes N(%)	No N(%)	Adj OR (95% CI)	Yes N(%)	No N(%)	AdjOR (95% CI)	Yes N(%)	No N(%)	AdjOR (95% CI)	Yes N(%)	No N(%)	AdjOR (95% CI)
1 (Mid Western Region)	Control	194 (66.4)	98 (33.6)	Ref	284 (97.3)	8 (2.7)	Ref	27 (9.2)	265 (90.8)	Ref	20 (6.8)	272 (93.2)	Ref	1 (0.3)	291 (99.7)	Ref	290 (99.3)	2 (0.7)	Ref	2 (0.7)	290 (99.3)	Ref
	Intervention	2318 (54.1)	1966 (45.9)	0.59 (0.46-0.76)	4252 (99.3)	30 (0.7)	3.99 (1.81-8.80)	1525 (35.6)	2759 (64.4)	5.45 (3.65-8.14)	1171 (27.3)	3113 (72.7)	5.12 (3.24-8.11)	42 (1.0)	4242 (99.0)	2.81 (0.39-20.5)	4259 (99.4)	25 (0.6)	1.14 (0.27-4.84)	12 (0.3)	4270 (99.7)	0.38 (0.08-1.70)
3 (Bharatpur)	Control	751 (23.7)	2418 (76.3)	Ref	2945 (93.7)	198 (6.3)	Ref	863 (27.2)	2306 (72.8)	Ref	871 (27.5)	2298 (72.5)	Ref	102 (3.2)	3067 (96.8)	Ref	3057 (96.5)	112 (3.5)	Ref	1285 (40.9)	1858 (59.1)	Ref
	Intervention	4971 (52.3)	4527 (47.7)	3.57 (3.26-3.91)	9224 (97.2)	265 (2.8)	2.34 (1.94-2.83)	5708 (60.1)	3790 (39.9)	4.07 (3.72-4.45)	5091 (53.6)	4407 (46.4)	3.08 (2.82-3.37)	63 (0.7)	9435 (99.3)	0.20 (0.15-0.27)	9421 (99.2)	77 (0.8)	4.54 (3.39-6.09)	174 (1.8)	9315 (98.2)	0.03 (0.02-0.03)
4 (Seti Zonal)	Control	919 (41.3)	1304 (58.7)	Ref	1959 (88.2)	261 (11.8)	Ref	421 (18.9)	1802 (81.1)	Ref	867 (39.0)	1356 (61.0)	Ref	37 (1.7)	2186 (98.3)	Ref	2180 (98.1)	43 (1.9)	Ref	1623 (73.1)	597 (26.9)	Ref
	Intervention	3778 (58.9)	2636 (41.1)	2.07 (1.87-2.28)	6107 (95.3)	303 (4.7)	2.74 (2.30-3.26)	3056 (47.6)	3358 (52.4)	3.93 (3.50-4.42)	3658 (57.0)	2756 (43.0)	2.11 (1.92-2.33)	35 (0.5)	6379 (99.5)	0.32 (0.20-0.50)	6331 (98.7)	83 (1.3)	1.53 (1.05-2.21)	5814 (90.7)	596 (9.3)	3.59 (3.16-4.07)
6 (Koshi Zonal)	Control	2777 (85.6)	468 (14.4)	Ref	2190 (67.8)	1040 (32.2)	Ref	2327 (71.7)	918 (28.3)	Ref	2606 (80.3)	639 (19.7)	Ref	9 (0.3)	3236 (99.7)	Ref	3219 (99.2)	26 (0.8)	Ref	495 (15.3)	2735 (84.7)	Ref
	Intervention	3886 (90.7)	398 (9.3)	1.83 (1.57-2.12)	3148 (73.7)	1124 (26.3)	1.33 (1.20-1.47)	2841 (66.3)	1443 (33.7)	0.82 (0.74-0.90)	2859 (66.7)	1425 (33.3)	0.53 (0.48-0.60)	32 (0.7)	4252 (99.3)	2.59 (1.21-5.53)	4252 (99.3)	32 (0.7)	0.99 (0.58-1.69)	256 (6.0)	4016 (94.0)	0.36 (0.31-0.43)

For peer review only

7 (Rapti Subregional)	Control	785 (31.8)	1686 (68.2)	Ref	995 (40.3)	1473 (59.7)	Ref	569 (23.0)	1902 (77.0)	Ref	1108 (44.8)	1363 (55.2)	Ref	17 (0.7)	2454 (99.3)	Ref	2424 (98.1)	47 (1.9)	Ref	22 (0.9)	2446 (99.1)	Ref
	Intervention	271 (15.5)	1473 (84.5)	0.39 (0.34-0.46)	266 (15.3)	1478 (84.7)	0.27 (0.23-0.31)	96 (5.5)	1648 (94.5)	0.19 (0.15-0.24)	875 (50.2)	869 (49.8)	1.23 (1.09-1.39)	17 (1.0)	1727 (99.0)	1.42 (0.72-2.80)	1732 (99.3)	12 (0.7)	2.86 (1.51-5.42)	29 (1.7)	1715 (98.3)	1.97 (1.13-3.46)
9 (Lumbini zonal)	Control	5512 (87.5)	791 (12.5)	Ref	4445 (71.0)	1813 (29.0)	Ref	4032 (64.0)	2271 (36.0)	Ref	4378 (69.5)	1925 (30.5)	Ref	73 (1.2)	6230 (98.8)	Ref	6268 (99.4)	35 (0.6)	Ref	183 (2.9)	6075 (97.1)	Ref
	Intervention	4242 (88.3)	564 (11.7)	1.05 (0.94-1.18)	2739 (57.1)	2058 (42.9)	0.54 (0.50-0.58)	2830 (58.9)	1976 (41.1)	0.80 (0.74-0.87)	2987 (62.2)	1819 (37.8)	0.72 (0.66-0.78)	17 (0.4)	4789 (99.6)	0.31 (0.18-0.52)	4764 (99.1)	42 (0.9)	0.63 (0.40-0.98)	42 (0.9)	4755 (99.1)	0.30 (0.21-0.42)
10 (Bheri Zonal)	Control	1547 (40.2)	2305 (59.8)	Ref	3621 (94.1)	228 (5.9)	Ref	1089 (28.3)	2763 (71.7)	Ref	1272 (33.0)	2580 (67.0)	Ref	8 (0.2)	3844 (99.8)	Ref	3842 (99.7)	10 (0.3)	Ref	13 (0.3)	3836 (99.7)	Ref
	Intervention	1204 (51.6)	1130 (48.4)	1.62 (1.46-1.80)	1146 (49.3)	1179 (50.7)	0.61 (0.05-0.07)	386 (16.5)	1948 (83.5)	0.51 (0.45-0.58)	373 (16.0)	1961 (84.0)	0.39 (0.34-0.45)	9 (0.4)	2325 (99.6)	1.88 (0.72-4.87)	2315 (99.2)	19 (0.8)	0.32 (0.15-0.69)	1 (0.0)	2324 (100.0)	0.13 (0.02-0.96)
All	Control	12485 (57.9)	9070 (42.1)	Ref	16439 (76.6)	5021 (23.4)	Ref	9328 (43.3)	12227 (56.7)	Ref	11122 (51.6)	10433 (48.4)	Ref	247 (1.1)	21308 (98.9)	Ref	21280 (98.7)	275 (1.3)	Ref	3623 (16.9)	17837 (83.1)	Ref
	Intervention	20670 (62.0)	12694 (38.0)	1.20 (1.16-1.25)	26882 (80.7)	6437 (19.3)	1.26 (1.21-1.31)	16442 (49.3)	16922 (50.7)	1.28 (1.24-1.33)	17014 (51.0)	16350 (49.0)	0.98 (0.95-1.01)	215 (0.6)	33149 (99.4)	0.55 (0.46-0.66)	33074 (99.1)	290 (0.9)	1.49 (1.27-1.76)	6328 (19.0)	26991 (81.0)	1.12 (1.07-1.17)

For peer review only

For peer review only

CONSORT 2010 checklist of information to include when reporting a randomised trial*

Section/Topic	Item No	Checklist item	Reported on page No
**Title and abstract**			
	1a	Identification as a randomised trial in the title	1
	1b	Structured summary of trial design, methods, results, and conclusions (for specific guidance see CONSORT for abstracts)	2
**Introduction**			
Background and objectives	2a	Scientific background and explanation of rationale	4
	2b	Specific objectives or hypotheses	5
**Methods**			
Trial design	3a	Description of trial design (such as parallel, factorial) including allocation ratio	5
	3b	Important changes to methods after trial commencement (such as eligibility criteria), with reasons	N/A
Participants	4a	Eligibility criteria for participants	6
	4b	Settings and locations where the data were collected	5
Interventions	5	The interventions for each group with sufficient details to allow replication, including how and when they were actually administered	6
Outcomes	6a	Completely defined pre-specified primary and secondary outcome measures, including how and when they were assessed	7-8
	6b	Any changes to trial outcomes after the trial commenced, with reasons	N/A
Sample size	7a	How sample size was determined	9
	7b	When applicable, explanation of any interim analyses and stopping guidelines	N/A
Randomisation:			
Sequence generation	8a	Method used to generate the random allocation sequence	N/A
	8b	Type of randomisation; details of any restriction (such as blocking and block size)	N/A
Allocation concealment mechanism	9	Mechanism used to implement the random allocation sequence (such as sequentially numbered containers), describing any steps taken to conceal the sequence until interventions were assigned	N/A
Implementation	10	Who generated the random allocation sequence, who enrolled participants, and who assigned participants to interventions	N/A
Blinding	11a	If done, who was blinded after assignment to interventions (for example, participants, care providers, those	N/A

For peer review only

For peer review only - http://bmjopen.bmj.com/site/about/guidelines.xhtml

		assessing outcomes) and how	
	11b	If relevant, description of the similarity of interventions	N/A
Statistical methods	12a	Statistical methods used to compare groups for primary and secondary outcomes	9
	12b	Methods for additional analyses, such as subgroup analyses and adjusted analyses	9
**Results**			
Participant flow (a diagram is strongly recommended)	13a	For each group, the numbers of participants who were randomly assigned, received intended treatment, and were analysed for the primary outcome	9-10
	13b	For each group, losses and exclusions after randomisation, together with reasons	9-10
Recruitment	14a	Dates defining the periods of recruitment and follow-up	7
	14b	Why the trial ended or was stopped	N/A
Baseline data	15	A table showing baseline demographic and clinical characteristics for each group	17
Numbers analysed	16	For each group, number of participants (denominator) included in each analysis and whether the analysis was by original assigned groups	10-11
Outcomes and estimation	17a	For each primary and secondary outcome, results for each group, and the estimated effect size and its precision (such as 95% confidence interval)	10-11
	17b	For binary outcomes, presentation of both absolute and relative effect sizes is recommended	10-11
Ancillary analyses	18	Results of any other analyses performed, including subgroup analyses and adjusted analyses, distinguishing pre-specified from exploratory	10-11
Harms	19	All important harms or unintended effects in each group (for specific guidance see CONSORT for harms)	N/A
**Discussion**			
Limitations	20	Trial limitations, addressing sources of potential bias, imprecision, and, if relevant, multiplicity of analyses	13
Generalisability	21	Generalisability (external validity, applicability) of the trial findings	13
Interpretation	22	Interpretation consistent with results, balancing benefits and harms, and considering other relevant evidence	11-13
**Other information**			
Registration	23	Registration number and name of trial registry	14
Protocol	24	Where the full trial protocol can be accessed, if available	14
Funding	25	Sources of funding and other support (such as supply of drugs), role of funders	14

*We strongly recommend reading this statement in conjunction with the CONSORT 2010 Explanation and Elaboration for important clarifications on all the items. If relevant, we also recommend reading CONSORT extensions for cluster randomised trials, non-inferiority and equivalence trials, non-pharmacological treatments, herbal interventions, and pragmatic trials. Additional extensions are forthcoming: for those and for up to date references relevant to this checklist, see www.consort-statement.org.

For peer review only - http://bmjopen.bmj.com/site/about/guidelines.xhtml

BMJ Open

Effect of a perinatal care quality improvement package on patient satisfaction: a secondary outcome analysis of a cluster randomized control trial

Journal:	*BMJ Open*
Manuscript ID	bmjopen-2021-054544.R1
Article Type:	Original research
Date Submitted by the Author:	07-Apr-2022
Complete List of Authors:	Brunell, Olivia; Uppsala University, Department of Women's and Children's Health<br>Chaulagain, Dipak; Uppsala University, Department of Women's and Children's Health<br>KC, Ashish; Uppsala University, Department of Women's and Children's Health<br>Bergström, Anna; Uppsala University, Department of Women's and Children's Health<br>Målqvist, Mats; Uppsala University, Department of Women's and Children's Health
**Primary Subject Heading**:	Global health
Secondary Subject Heading:	Paediatrics
Keywords:	PAEDIATRICS, NEONATOLOGY, Quality in health care < HEALTH SERVICES ADMINISTRATION & MANAGEMENT

SCHOLARONE™
Manuscripts

I, the Submitting Author has the right to grant and does grant on behalf of all authors of the Work (as defined in the below author licence), an exclusive licence and/or a non-exclusive licence for contributions from authors who are: i) UK Crown employees; ii) where BMJ has agreed a CC-BY licence shall apply, and/or iii) in accordance with the terms applicable for US Federal Government officers or employees acting as part of their official duties; on a worldwide, perpetual, irrevocable, royalty-free basis to BMJ Publishing Group Ltd ("BMJ") its licensees and where the relevant Journal is co-owned by BMJ to the co-owners of the Journal, to publish the Work in this journal and any other BMJ products and to exploit all rights, as set out in our licence.

The Submitting Author accepts and understands that any supply made under these terms is made by BMJ to the Submitting Author unless you are acting as an employee on behalf of your employer or a postgraduate student of an affiliated institution which is paying any applicable article publishing charge ("APC") for Open Access articles. Where the Submitting Author wishes to make the Work available on an Open Access basis (and intends to pay the relevant APC), the terms of reuse of such Open Access shall be governed by a Creative Commons licence – details of these licences and which Creative Commons licence will apply to this Work are set out in our licence referred to above.

Other than as permitted in any relevant BMJ Author's Self Archiving Policies, I confirm this Work has not been accepted for publication elsewhere, is not being considered for publication elsewhere and does not duplicate material already published. I confirm all authors consent to publication of this Work and authorise the granting of this licence.

For peer review only

Effect of a perinatal care quality improvement package on patient satisfaction: a secondary outcome analysis of a cluster randomized control trial

Olivia Brunell[1], Dipak Chaulagain[1], Ashish KC[1], Anna Bergström[1], Mats Målqvist[1]

[1] Department of Women's and Children's Health, Uppsala University

Corresponding author

Olivia Brunell, Department of Women's and Children's Health, Uppsala University,

Sjukhusvägen 7, 753 09 Uppsala, Sweden

e-mail: olivia.brunell@kbh.uu.se

phone: +46 8611 00 00

Keywords

Neonatology, Perinatal care, Quality Improvement, Patient Satisfaction

Word count 3674

For peer review only

ABSTRACT

Objective This study aimed to investigate the effect of a quality improvement (OI) package on patient satisfaction of perinatal care.

Design Secondary analysis of a stepped-wedge cluster randomized controlled trial. Participating hospitals were randomized by size into four different wedges.

Setting 12 secondary-level public hospitals in Nepal.

Participants Women who gave birth in the hospitals at a gestational age of ≥22weeks, with fetal heart sound at admission. Adverse outcomes were excluded. One hospital was excluded due to data incompleteness and four low-volume hospitals due to large heterogeneity. The final analysis included 54,919 women.

Intervention Hospital management was engaged and facilitators were recruited from within hospitals. Available perinatal care was assessed in each hospital, followed by a bottle-neck analysis workshop. A three-day training in essential newborn care was carried out for health workers involved in perinatal care, and a set of quality improvement tools were introduced to be used in every day practice (skill-checks, self- assessment checklists, scoreboards, and weekly Plan-Do-Study-Act-meetings). Refresher training after 6 months.

For peer review only

Outcome measure Women's satisfaction with delivery care.

Results The likelihood of women being overall satisfied with delivery care increased after the intervention, [aOR 1.66(1.59-1.73)]. The proportions of overall satisfaction were however low (control 58%, intervention 62%). Women were more likely to be satisfied with education and information from health workers after intervention [aOR 1.34(1.29-1.40)] and to have been treated with dignity and respect [aOR 1.81(1.52-2.16)]. The likelihood of having experienced abuse during the hospital stay decreased [aOR: 0.42(0.34-.051)] and of being satisfied with the level of privacy increased [aOR 1.14, 95%(1.09-1.18)].

Conclusions Improvements in patient satisfaction were indicated after the introduction of a QI-package on perinatal care. We recommend further studies on which aspects of care are most important to improve women's satisfaction of perinatal care in hospitals in Nepal.

Trial registration ISRCTN30829654

For peer review only

ARTICLE SUMMARY

Strengths and limitations of this study

- This secondary analysis focuses on patients' experience of care, an important dimension of quality improvement which is often overlooked when evaluating quality improvement efforts

For peer review only - http://bmjopen.bmj.com/site/about/guidelines.xhtml

- The large sample size from different hospitals increases representativeness of the sample and generalizability of our results, while external validity is strengthened by the use of the WHO framework
- The stepped-wedge cluster-randomization allowed us to eventually roll out the intervention at all sites, while mimicking a randomized-controlled trial
- The exclusion of one high-volume hospital and the four low- volume hospitals, deviates from the original stepped-wedged design and should be considered when interpreting the results
- We measured the WHO domains of experience of care and no other factors that are also known to affect satisfaction, such as waiting times, overcrowding and physical resources in the health facility

For peer review only

For peer review only - http://bmjopen.bmj.com/site/about/guidelines.xhtml

INTRODUCTION

In 2017 an estimated 2,5 million children died in the neonatal period whereof about 1 million newborn died during their first day of life[1]. Though an increasing number of women are giving birth in health facilities it has not ensured improved health care outcomes[2]. The quality of perinatal care provided in health facilities needs improvement to end preventable mortality[2-4]. Both Quality of Care (QoC) and approaches to its assessment are complex [5]. The definition of QoC by the World Health Organization (WHO) is "The extent to which health care services provided to individuals and patient populations improve desired health outcomes" and to achieve this, health care must be safe, effective, timely, efficient, equitable and people-centered. Thus, health care must not only provide evidence based clinical care, but by being people-centered, the care must also be provided considering individuals preferences, needs and values and the culture of their communities[6]. This is important as patients have the right to be treated with dignity and respect, but also because people-centered care is associated with improved health care utilization and health outcomes[7]. In 2016, WHO developed a framework and standards with accompanying quality measures for the quality of maternal and newborn health care. In this framework, QoC is divided into two interlinked dimensions; (1) *Provision of clinical care* by health workers, and (2) patients' *Experience of care*[8] (Figure 1). *Experience of care* is a people-centered measure, a process indicator which reflects the interactions that patients have with the healthcare system[9]. It is composed of three major domains; effective communication, respect and dignity, and emotional support[6]. Patient satisfaction, together with other people-centered outcomes, is an outcome of *Experience of car*e. [9-11].

For peer review only - http://bmjopen.bmj.com/site/about/guidelines.xhtml

In 2013 the research group conducted a study of the implementation of a quality improvement (QI) package for neonatal resuscitation in a tertiary hospital in Nepal. In this study the Helping Babies Breathe (HBB) program, developed by The American Academy of Pediatrics to improve health workers' performance on basic resuscitation[12, 13], was implemented. In addition to HBB-training, the intervention included QI-components, aiming to continuously reinforce the HBB protocol throughout the intervention period and to motivate both hospital management and individual health workers to be engaged in improvement.

The study, Helping Babies Breath-Quality Improvement Cycle (HBB-QIC), showed improvement in health workers adherence to neonatal resuscitation protocols and a large decrease in intrapartum still births and first-day neonatal mortality [14]. These positive results are more pronounced than in previous studies of implementation of the HBB-program [15, 16] and can be due to the addition of the QI components.

However, there were no significant change of overall in-hospital perinatal mortality, which indicates that efforts are also needed to improve the continued postnatal care[17]. Based on the findings from HBB-QIC, a scale-up was developed by the research group in collaboration with Ministry of Health and Population in Nepal. This is called Nepal Perinatal Quality Improvement Project (NePeriQIP) and aims to improve quality of perinatal care through increased clinical skills and knowledge in essential newborn care (ENC) and establish a structure for continuous quality improvement[18]. The primary objective of the NePeriQIP trial was to evaluate impact on intrapartum mortality (intrapartum stillbirth and first-day mortality), and the results showed a significant reduction in intrapartum-related deaths (intrapartum stillbirths and first day mortality) in the intervention period (aOR, 0.79; 95% CI, 0.69–0.92)[19]. The aim of this study is to evaluate the effect of NePeriQIP on the secondary outcome *patient satisfaction*.

For peer review only - http://bmjopen.bmj.com/site/about/guidelines.xhtml

METHODS

Trial design

A stepped-wedge cluster-randomized design was used[18], where 12 hospitals were randomized by size into four wedges. Each wedge included one high-volume (>8000 deliveries a year), one medium-volume (>3000 deliveries a year) and one low-volume (>1000 deliveries a year) hospital. Each cluster had a control and an intervention period. During the first three months of the study period no intervention activities took place at any of the hospitals (baseline period). NePeriQIP was then introduced to one wedge with three hospitals at a time, with a three months delay between wedges, and eventually all hospitals were exposed to the intervention (Figure 2).

Setting

The selected 12 hospitals filled the criteria of having >1000 deliveries per year and being a referral center for maternal and newborn care. Although most of the hospitals were located in the flatlands, they were diverse in relation to ethnicity, language and religion. All hospitals provided vaginal and cesarian deliveries, and had access to neonatal resuscitation services at birth. Skilled birth-attendants (obstetricians, medical doctors, senior nurses or senior nurse midwives) led the labor units. Medical doctors led the pediatric units where sick newborns were managed in the low-volume hospitals. Pediatricians led the specialized sick newborn care units in the medium and high-volume hospitals. The intrapartum related mortality rate (intrapartum stillbirth and first day neonatal mortality) ranged from 9 to 31 per 1,000 births at the hospitals (mean 13/1000 births), during the baseline period[19].

For peer review only - http://bmjopen.bmj.com/site/about/guidelines.xhtml

Participants

All pregnant women who gave birth at any of the 12 hospitals after the baseline period, who gave their oral and written consent, who were at gestational age of 22 weeks or more and who had fetal heart sound at admission were eligible for inclusion in the study. Women who experienced stillbirths, neonatal deaths, malformations and complications during childbirth were excluded as these events could bias patient satisfaction.

The intervention

The development of the QI-package was guided by the integrated-Promoting Action on Research Implementation in Health Services (iPARIHS) framework [20]. Ministry of Health, together with the research team, developed an implementation guideline for the implementation process. In each hospital, the QI-package was introduced in the following steps: First, the hospital management was oriented on the QI-package. They appointed in-hospital facilitators from among pediatricians, medical officers and nurses to facilitate the implementation, supported by the study team. The number of facilitators depended on the size of the hospital: two from low-volume, three from medium-volume and four from high-volume-hospitals. The facilitators participated in a seven-day training on the QI-package, provided by the study team. Following the training, the QI facilitators assessed service readiness and availability in their respective hospital, using a checklist developed by the team, and a bottleneck analysis workshop was organized based on that. After these initial steps, a three-day on-site training for all health staff involved in perinatal care was organized by the facilitators and the study team. The training consisted of education and skills-training in ENC and introduction of a set of QI-tools. The ENC component included resuscitation and immediate newborn care (thorough drying, skin-to-skin contact, delayed clamping of the cord and early initiation of breastfeeding) and were taught using the HBB training manual version

For peer review only

one[12]. In addition, lectures were given on breastfeeding, infection prevention and kangaroo mother care for low birth-wight newborns. The QI- tools included daily bag-and-mask ventilation skill check on an HBB-mannequin (the low-cost simulator Laerdal® NeoNatalie), checklist for preparations before every delivery, self-assessment checklist of performed resuscitation, and use of score-boards comprising major indicators on neonatal resuscitation. In addition to these tools a structured model for quality improvement, Plan-Do-Study-Act (PDSA)-cycle , was used aiming to identify and act upon local problems [21]. After the training, the QI facilitators initiated weekly PDSA-meetings as part of routine work at all units, involving health workers related to perinatal care. The duration of each meeting was about half an hour. A one-day refresher training in HBB was held for health workers after six months.

Conceptual framework

The WHO conceptual framework on QoC (Figure 1)[6] and the WHO-document "Standards for improving quality of maternal and newborn care in health facilities"[8] were used to guide data collection and analyzes for this sub-study. In the framework, QoC consists of the two inter-linked dimensions 'Provision of care' and 'Experience of care'. Experience of care contains three domains; *effective communication*, *preservation of respect and dignity*, and *emotional support*. Though NePeriQIP focus on clinical provision of care, a successful implementation where health workers are more engaged in the quality of perinatal care could generate better experiences among patients and manifest in patient satisfaction.

Data collection and variables

The study period followed the Nepali calendar and was initiated on June 2017 and ran for 15 months up until October 2018. Data was collected through structured interviews with all

For peer review only - http://bmjopen.bmj.com/site/about/guidelines.xhtml

included mothers upon discharge, throughout the study period. An independent data collection team was established at each hospital and a structured questionnaire developed by the study team and piloted on beforehand in a hospital later not included in the study, was used to collect the data. After completion of the data collection forms, they were sent weekly to the central research office in Kathmandu, where they were entered into a Census and Survey Processing System database by a team of independent data entry officers.

The collected data from exit interviews was not accessible for the local teams during the intervention. Results will be shared with the local teams by the published paper.

The outcome of this study was Patient satisfaction. This was measured through *Overall satisfaction* and the three dimensions of *Experience of care* (effective communication, respect and dignity, and emotional support) in the WHO Quality of Care framework for maternal and newborn health [6].

Overall satisfaction was measured as an aggregate score of two questions, and to be considered satisfied with services received, mothers needed to respond favorably to both questions.

(1) *Overall, how satisfied are you with the services?*

(Recorded on a Likert scale of 1 to 5. 1= very dissatisfied, 2= dissatisfied, 3= neither, 4= satisfied, 5= very satisfied. Scores were dichotomized, with an answer of 4 or more being set as satisfied).

(2) *Would you recommend a friend to deliver at this hospital?*

(recorded yes/ no)

For peer review only - http://bmjopen.bmj.com/site/about/guidelines.xhtml

Experience of care was investigated through a set of questions suggested as quality measures of experience of care in the WHO-document “Standards for improving quality of maternal and newborn care in health facilities”[8].

(1) Patient communication

Were you adequately informed by the health care worker about examinations, actions and decisions taken for your care throughout the hospital stay?

(recorded yes/no)

Are you satisfied with health education and information received from health care providers?

(Recorded on a Likert scale of 1 to 5. 1= very dissatisfied, 2= dissatisfied, 3= neither, 4= satisfied, 5= very satisfied. Scores were dichotomized, with an answer of 4 or more being set as satisfied).

(2) Respect and dignity

Are you satisfied with the degree of privacy during your stay in labor and child birth areas?

(Recorded on a Likert scale of 1 to 5. 1= very dissatisfied, 2= dissatisfied, 3= neither, 4= satisfied, 5= very satisfied. Scores were dichotomized, with an answer of 4 or more being set as satisfied).

Where you or your newborn physically, verbally or sexually abused during labor or childbirth or after birth?

(recorded yes/no)

Were you treated with respect and was your dignity preserved during your stay at the hospital?

For peer review only - http://bmjopen.bmj.com/site/about/guidelines.xhtml

(recorded yes/no)

(3) Emotional support

Did you have a companion of your choice during labor and childbirth?

(recorded yes/no)

Statistical methods

There was no a priori estimation of sample size for this sub-study, it was calculated for the primary outcome of the larger NePeriQIP-trial, intrapartum mortality[19] . Given that intrapartum mortality is a rare outcome, this rendered a large sample-size, which allowed for the detection of small differences in the outcomes of interest for the present study. Pearson's Chi-Squared test was used to analyze background characteristics of participants in the control and intervention groups, performed with Statistical Package for Social Sciences (SPSS) version 25.0. Change in satisfaction and experiences of care between the control and intervention groups was analyzed by generalized linear mixed models (GLMM) using the software R (version 3.4.0). Analyses were performed with R package lme4. Adjustments were made for structural factors (cast/ethnicity and educational level) as these could be ground for discrimination and altered treatment. Intra-cluster correlations coefficients (ICC) for each outcome were calculated. Initial analyses showed high ICC for all outcome variables, making the results inconclusive. Further analysis of the data by hospital displayed that the low-volume hospitals showed large heterogeneity. As a consequence, the final GLMM analysis was performed on the medium and high-volume hospitals. Analysis of change before and after intervention at each hospital was performed with binary logistic regression of satisfaction and experience of care (adjusted for caste/ethnicity and education level) with

For peer review only

Statistical Package for Social Sciences (SPSS) version 25.0. Significance level of 95% was used.

Ethical considerations

Ethical approval was obtained from the Nepal Health Research Council (ref 26–2017). The data used in this study was collected through face-to-face interviews after obtaining written informed consent from the participants. The identity of the participants was coded after data collection, and the code key was stored in a safe environment that only the project management had access to.

Patient and public involvement

Patients were not directly involved in developing this study, however it was planned and conducted in collaboration with Ministry of Health in Nepal.

RESULTS

A total number of 65,895 women were eligible for inclusion in this study, whereof 946 women were excluded based on exclusion criteria. Due to data incompleteness from one of the hospitals, another 5,366 women were excluded, leaving 59,583 women available for analysis. Initial analysis revealed that low-volume hospitals showed large heterogeneity and differed from the medium and high-volume hospitals. As a consequence of this finding, the final GLMM analysis was performed on the medium and high-volume hospitals where 92% of deliveries took place, n= 54,919 (Figure 3).

Characteristics

For peer review only

A majority of the women had an education higher than primary (69%) and belonged to an advantageous cast (67%). About half of the women were first-time mothers (51%) but few were adolescents (7.6%) and the coverage of antenatal care was high (80% had four antenatal care visits before delivery). The cesarean section rate was 19% and most of them (73%) were emergency cesarean sections. The proportion of preterm deliveries were 13% and babies with low birth weight were 14% (Table 1).

Outcomes

Women were more likely to be satisfied with care after the intervention than before, adjusted Odds Ratio (aOR) 1.66 [CI: 1.59-1.73, ICC: 0.275]). The results varied between hospitals (aOR 0.39-3.57) (Table S1) and the overall proportion of satisfaction was low. Prior to the intervention a proportion of 58% of the women were satisfied, which increased to 62% after (Table 2).

The domains of experience of care were affected in a positive direction by the intervention. Women were more likely to be *satisfied with education and information* from health workers after implementation (aOR 1.34 [CI: 1.29-1.40], ICC: 0.167) as well as more likely to state that they had been *treated with preserved dignity and respect* (aOR: 1.81 [CI: 1.52-2.16], ICC: 0.063]). The likelihood of *Experience abuse during the hospital stay* decreased after the implementation (aOR: 0.42 [CI: 0.34-.051, ICC: 0.082]) and the likelihood of being *satisfied with the level of privacy* increased (aOR 1.14 [CI: 1.09-1.18, ICC: 0.136]) (Table 2). Two measures seemed to have been affected in a negative direction after the intervention: the likelihood of being *adequately informed about examinations, actions and decisions taken for their care* (aOR 0.55 [CI: 0.53-0.58, ICC: 0.507] and *having a companion of choice during labor* (aOR 0.27 [CI:0.25-0.29, ICC: 0.687]. These two measures, however, displayed

For peer review only

relatively high ICC in the GLMM model, rendering the adjusted odds ratios to be inconclusive. (Table 2).

A very high proportion of women stated that they were treated with dignity and respect (before intervention 98.7%, after 99.1%) and very few had experienced abuse during their hospital stay (1.1% before intervention and 0.6% after). The proportion of women who stated that they were adequately informed about their care were 76.3% before intervention and 80.6% after. However, not many women had a companion of choice during labor (16.8% before and 19.0% after), and the proportions of women who were satisfied with the information and education from health workers, and with the level of privacy, were also low (43.3% before and 49.3% after, 51.6% before and 51.0% after, respectively) (Table 2).

DISCUSSION

The quality of perinatal care in health facilities needs to be improved to save newborn lives [2-4]. Measuring patient satisfaction is important in several aspects to improve quality of care; It can reveal gaps in the health system to address, it creates accountability among health care providers and stakeholders, it is useful to guide and evaluate QI efforts, and in addition satisfaction is known to affects patients care-seeking behavior and compliance[9, 22-24]. Our study shows that after the introduction of the QI-package of NePeriQIP, the likelihood that women were satisfied with perinatal care increased. Satisfaction is an outcome measure of experience of care [9], and in our study the people-centered quality measures of experience of care suggested by WHO were affected in a positive direction, along with satisfaction. The exact mechanisms behind our results are not clear as the intervention did not specifically focus on people-centered skills. However, besides that NePeriQIP focused on improvement of

For peer review only - http://bmjopen.bmj.com/site/about/guidelines.xhtml

provision of care through training, it also included QI-tools to strengthen professional attitudes among health workers and sustain change in practice. Self-evaluation, insight in the ongoing progress and involvement in problem-solving promote a higher level of learning which can create a deeper understanding among health workers and make them pay more attention to what they are doing and why [25]. This can improve their capacity to inform and educate patients, to handle stressful situations and provide more time and engagement to attend to interpersonal interactions with patients. With increased knowledge and continuous discussions concerning care of the mothers and newborn, healthcare workers might also get more sensitized and empathic towards their patients. A recent implementation study conducted in India, also adopting the PDSA-approach and facilitation to improve quality of care, could similarly to our study show improvements in satisfaction among recently delivered women [26].

Satisfaction is a complex phenomenon influenced by several determinants, and we cannot conclude that the improvement in satisfaction is explained solely by improvement in the measures of experience of care that we have studied. Other known determinants of satisfaction are structural elements (i.e., cleanliness of the health facility and availability of human recourses, medicines and supplies) and outcome related determinants such as health status of the mother and newborn[27]. Nevertheless, it is previously shown that the inter-personal components of experience of care dominate satisfaction with maternity care in Nepal and other low-income countries[27, 28].

Though we could see an increased satisfaction after the intervention, the proportion of satisfied women both before and after was low. This despite that women tend to report more satisfied with care if interviewed early after childbirth, biased by social desirability and the joy of having a healthy baby [29] and previous studies of satisfaction in Nepal and India has

For peer review only - http://bmjopen.bmj.com/site/about/guidelines.xhtml

shown higher levels [26, 30, 31]. We do not know why the women were not more satisfied with care but some factors can be considered. First, we sought to reduce the influence of social desirability by our definition of satisfaction (overall satisfaction combined with whether to recommend the facility to a friend). In addition, other determinants that was not the scope of our study, could be of importance. Other studies of satisfaction with perinatal care in Nepal has shown that having a protected waiting area and having an opportunity to ask questions are positively associated with patient satisfaction, while over-crowding and long waiting times has been shown to increase the likelihood of dissatisfaction[28, 31].

We adjusted for the structural determinants cast/ethnicity and educational level, which can be grounds for discrimination, altered treatment and outcomes. The intermediate determinants displayed in Table 1 were not considered grounds for discrimination and thus not included in the further analysis. Given the complexity of the stepped-wedge design, no further multi-level analyses were performed.

The main strengths of our study are the large sample and the stepped-wedge cluster-randomization. The latter allowed us to eventually roll out the intervention at all sites, while mimicking a randomized-controlled trial. Our study was also guided by WHO framework which strengthens external validity.

There are some limitations to our study worth mentioning. We excluded women with adverse outcomes since this could over shadow the experience of care and skew our results. We had to exclude one high-volume hospital due to data incompleteness and the four low-volume hospitals due to initial analysis showing high ICC. This deviates from the original stepped-wedged design and should be considered when interpreting the results. In this study we only focused on measuring the WHO domains of experience of care and no other factors that are also known to affect satisfaction, such as waiting times, overcrowding, patients' expectations,

For peer review only - http://bmjopen.bmj.com/site/about/guidelines.xhtml

provision of medical care or physical resources in the health facility[28, 32]. A more in-depth understanding of the women's satisfaction could have been achieved by adding qualitative interviews.

CONCLUSION

Measuring patient satisfaction is an important aspect of quality-of-care development and evaluation. We found that after the introduction of a QI-package (NePeriQIP), women giving birth in health facilities were significantly more likely to be satisfied with care. Given the large sample size and the modest increase in the proportion of overall stated satisfaction, the results should however be interpreted with caution. As satisfaction with perinatal care in Nepal still needs improvement, we recommend further studies on which aspects of care that are of most importance to improve patient satisfaction.

ACKNOWLEDGEMENTS

The authors would like to thank the management and health care staff of all participating hospitals as well as facilitators, mentors, data collectors, and the data management team at Golden Community. Thanks to Abhishek Gurung, Elisha Joshi, Sunil Gajurel, Prajwal Poudel and Asmita Paudel for facilitating the QI implementation process in hospitals. Special thanks to Omkar Basnet for excellent work in data management and to Regina Gurung for participating in managing the project. Lastly, we would like to express our gratitude towards all participating mothers.

For peer review only - http://bmjopen.bmj.com/site/about/guidelines.xhtml

ADDITIONAL INFORMATION

Author Contributions OB, MM, AB and AK conceptualized the study. AK and DC supervised data collection and management. OB and MM analyzed data and prepared the first draft, as well as the final version. All authors, reviewed, commented and approved the final version of the manuscript.

Trial registration number ISRCTN30829654

The full trial protocol "Scaling up quality improvement intervention for perinatal care in Nepal (NePeriQIP); study protocol of a cluster randomized trial" can be accessed at BMJ Global Health[18]

Funding This work was supported by Swedish Research Council (2016-05621), the Laerdal Foundation for Acute Medicine, Norway (40198), and Einhorn Family Foundation, Sweden. The funders had no role in study design, data collection and analysis, decision to publish, or preparation of the manuscript.

Competing interests There are no competing interests for any author.

Data sharing statement Deidentified participant data used in the analysis of this manuscript will be available upon request. The study protocol has been published [18].

REFERENCES

For peer review only - http://bmjopen.bmj.com/site/about/guidelines.xhtml

1. Hug L, Sharrow D, Zhong K, You D: **Levels & Trends in Child Mortality**. In.: United Nations Inter-agency Group for Child Mortality Estimation (UN IGME); 2018.
2. Scott KW, Jha AK: **Putting quality on the global health agenda**. *N Engl J Med* 2014, **371**(1):3-5.
3. Kruk M, Gage A, Joseph N, Danaei G, Garcia-Saisó S, Salomon JA: **Mortality due to low-quality health systems in the universal health coverage era: a systematic analysis of amenable deaths in 137 countries**. *The Lancet* 2018, **392**(10160):2203-2212.
4. Bhutta Z, Das JK, Bahl R, Lawn JE, Salam RA, Vinod K Paul VK, Sankar MJ, Blencowe H, Rizvi A, Chou VB *et al*: **Every Newborn: Can available interventions end preventable deaths in mothers, newborn babies, and stillbirths, and at what cost?** . *The Lancet* 2014.
5. Hanefeld J, Powell-Jackson T, Balabanova D: **Understanding and measuring quality of care: dealing with complexity**. *Bull World Health Organ* 2017, **95**(5):368-374.
6. Tuncalp, Were WM, MacLennan C, Oladapo OT, Gulmezoglu AM, Bahl R, Daelmans B, Mathai M, Say L, Kristensen F *et al*: **Quality of care for pregnant women and newborns-the WHO vision**. *BJOG* 2015, **122**(8):1045-1049.
7. Doyle C, Lennox L, Bell D: **A systematic review of evidence on the links between patient experience and clinical safety and effectiveness**. *BMJ Open* 2013, **3**(1).
8. WHO.: **Standards for improving quality of maternal and newborn care in health facilities**. In. Geneva: WHO Press; 2016.
9. Larson E, Sharma J, Bohren MA, Tuncalp O: **When the patient is the expert: measuring patient experience and satisfaction with care**. *Bull World Health Organ* 2019, **97**(8):563-569.

For peer review only

For peer review only - http://bmjopen.bmj.com/site/about/guidelines.xhtml

10. Donabedian A: **The Definition of Quality and Approaches to Its Assessment, vol 1: Explorations in Quality Assessment and Monitoring**. University of Michigan: Health Administration Press; 1980.

11. Cleary PD, McNeil BJ: **Patient satisfaction as an indicator of quality care**. *Inquiry* 1988, **25**(1):25-36.

12. Singhal N, Lockyer J, Fidler H, Keenan W, Little G, Bucher S, Qadir M, Niermeyer S: **Helping Babies Breathe: global neonatal resuscitation program development and formative educational evaluation**. *Resuscitation* 2012, **83**(1):90-96.

13. Niermeyer S: **From the Neonatal Resuscitation Program to Helping Babies Breathe: Global impact of educational programs in neonatal resuscitation**. *Semin Fetal Neonatal Med* 2015, **20**(5):300-308.

14. Kc A, Wrammert J, Clark RB, Ewald U, Vitrakoti R, Chaudhary P, Pun A, Raaijmakers H, Malqvist M: **Reducing Perinatal Mortality in Nepal Using Helping Babies Breathe**. *Pediatrics* 2016, **137**(6).

15. Msemo G, Massawe A, Mmbando D, Rusibamayila N, Manji K, Kidanto HL, Mwizamuholya D, Ringia P, Ersdal HL, Perlman J: **Newborn mortality and fresh stillbirth rates in Tanzania after helping babies breathe training**. *Pediatrics* 2013, **131**(2):e353-360.

16. Goudar SS, Somannavar MS, Clark R, Lockyer JM, Revankar AP, Fidler HM, Sloan NL, Niermeyer S, Keenan WJ, Singhal N: **Stillbirth and newborn mortality in India after helping babies breathe training**. *Pediatrics* 2013, **131**(2):e344-352.

17. Wrammert J, Kc A, Ewald U, Malqvist M: **Improved postnatal care is needed to maintain gains in neonatal survival after the implementation of the Helping Babies Breathe initiative**. *Acta Paediatr* 2017, **106**(8):1280-1285.

For peer review only

18. Kc A, Bergstrom A, Chaulagain D, Brunell O, Ewald U, Gurung A, Eriksson L, Litorp H, Wrammert J, Gronqvist E *et al*: **Scaling up quality improvement intervention for perinatal care in Nepal (NePeriQIP); study protocol of a cluster randomised trial**. *BMJ Glob Health* 2017, **2**(3):e000497.

19. Kc A, Ewald U, Basnet O, Gurung A, Pyakuryal SN, Jha BK, Bergstrom A, Eriksson L, Paudel P, Karki S *et al*: **Effect of a scaled-up neonatal resuscitation quality improvement package on intrapartum-related mortality in Nepal: A stepped-wedge cluster randomized controlled trial**. *PLoS Med* 2019, **16**(9):e1002900.

20. Harvey G, Kitson A: **PARIHS revisited: from heuristic to integrated framework for the successful implementation of knowledge into practice**. *Implement Sci* 2016, **11**:33.

21. Christoff P: **Running PDSA cycles**. *Curr Probl Pediatr Adolesc Health Care* 2018, **48**(8):198-201.

22. Al-Abri R, Al-Balushi A: **Patient satisfaction survey as a tool towards quality improvement**. *Oman Med J* 2014, **29**(1):3-7.

23. Quintana JM, Gonzalez N, Bilbao A, Aizpuru F, Escobar A, Esteban C, San-Sebastian JA, de-la-Sierra E, Thompson A: **Predictors of patient satisfaction with hospital health care**. *BMC Health Serv Res* 2006, **6**:102.

24. Bernhart MH, Wiadnyana IG, Wihardjo H, Pohan I: **Patient satisfaction in developing countries**. *Soc Sci Med* 1999, **48**(8):989-996.

25. Anderson L.W. KDR, Bloom B.S: **A Taxonomy for Learning, Teaching, and Assessing : a Revision of Bloom's Taxonomy of Educational Objectives / Editors, Lorin W. Anderson, David Krathwohl ; Contributors, Peter W. Airasian ... [et Al.]**. . New York: Longman; 2001.

For peer review only

26. Das MK, Arora NK, Dalpath SK, Kumar S, Kumar AP, Khanna A, Bhatnagar A, Bahl R, Nisar YB, Qazi SA *et al*: **Improving quality of care for pregnancy, perinatal and newborn care at district and sub-district public health facilities in three districts of Haryana, India: An Implementation study**. *PLoS One* 2021, **16**(7):e0254781.

27. Srivastava A, Avan BI, Rajbangshi P, Bhattacharyya S: **Determinants of women's satisfaction with maternal health care: a review of literature from developing countries**. *BMC Pregnancy Childbirth* 2015, **15**:97.

28. Mehata S, Paudel YR, Dariang M, Aryal KK, Paudel S, Mehta R, King S, Barnett S: **Factors determining satisfaction among facility-based maternity clients in Nepal**. *BMC Pregnancy Childbirth* 2017, **17**(1):319.

29. Lumley J: **Assessing satisfaction with childbirth**. *Birth* 1985, **12**(3):141-145.

30. Ministry of Health and Population (MOHP) [Nepal] HRaSDFH, and Nepal Health Sector Support Programme (NHSSP): **Service Tracking Survey 2013**. In. Kathmandu, Nepal: Ministry of Health and Population, HERD, and NHSSP, Ramsah Path, Kathmandu, Nepal; 2014.

31. Acharya S, S. Sharma, B. Dulal, K. Aryal: **Quality of Care and Client Satisfaction with Maternal Health Services in Nepal: Further Analysis of the 2015 Nepal Health Facility Survey. DHS Further Analysis Reports No. 112**. In. Rockville, Maryland, USA: ICF; 2018.

32. Bjertnaes OA, Sjetne IS, Iversen HH: **Overall patient satisfaction with hospitals: effects of patient-reported experiences and fulfilment of expectations**. *BMJ Qual Saf* 2012, **21**(1):39-46.

For peer review only

TABLES

Table 1. Characteristics of study participants in control and intervention group. Chi2-test to detect group differences before and after NePeriQIP intervention start (n_{tot}=59,583)

**Demographic characteristics**		**Total n (%)**	**Control n (%)**	**Intervention n (%)**	**p-value**
Structural determinants					
Mother's cast/ethnicity	relatively disadvantaged	19,667 (33.0)	8,537 (35.9)	11,130 (31.1)	<0.001*
	relatively advantaged	39,916 (67.0)	15,270 (64.1)	24,646 (68.9)	
Mother's education	Primary or less	18,591 (31.3)	7,572 (31.9)	11,019 (30.9)	0.006*
	Secondary or higher	40,829 (68.7)	16,139 (68.1)	24,690 (69.1)	
Intermediate determinants					
Adolescent mother (≤19 years)	No	55,061 (92.4)	22,026 (92.5)	33,035 (92.3)	0.415
	Yes	4,522 (7.6)	1,781 (7.5)	2,741 (7.7)	
Elder mother (>35 years)	No	59,012 (98.9)	23,667 (98.9)	35,345 (98.8)	0.082
	Yes	681 (1.1)	250 (1.1)	431 (1.2)	
Parity	Nulli	30,610 (51.4)	12,400 (52.1)	18,210 (50.9)	0.005*
	Multi	28,973 (48.6)	11,407 (47.9)	17,566 (49.1)	
>Four ANC visits	No	11,700 (19.9)	5,136 (21.9)	6,564 (18.5)	0.000*
	Yes	47,131 (80.1)	18,273 (78.1)	28,858 (81.5)	
Spontaneous vaginal	No	14,628 (23.3)	5,294 (22.2)	8,704 (24.3)	<0.001*
	Yes	45,585 (76.7)	18,513 (77.8)	27,072 (75.7)	
Elective CS	No	56,563 (94.9)	22,413 (94.1)	34,150 (95.5)	<0.001*

For peer review only - http://bmjopen.bmj.com/site/about/guidelines.xhtml

	Yes	3,020 (5.1)	1,394 (5.9)	1,626 (4.5)	
Emergency CS	No	51,559 (86.5)	20,846 (87.6)	30,713 (85.8)	<0.001*
	Yes	8,024 (13.5)	2,961 (12.4)	5,063 (14.2)	
Preterm	No	51,579 (86.6)	21,740 (91.3)	29,839 (83.4)	<0.001*
	Yes	8,004 (13.4)	2,067 (8.7)	5,937 (16.6)	
LBW	No	46,997 (86.2)	20,540 (87.4)	26,457 (85.3)	<0.001*
	Yes	7,524 (13.8)	2,949 (12.6)	4,575 (14.7)	
Sex of baby	Boy	32,132 (54.1)	12,771 (53.9)	19,361 (54.2)	0.391
	Girl	27,288 (45.9)	10,940 (46.1)	16,348 (45.8)	

For peer review only

Table 2. Binary logistic regression for full sample and GLMM analysis correcting for clustering (by hospital) displaying adjusted odds ratios. Chi2-test for group differences. (n=54,919).

					Generalized Linear Mixed Method (GLMM)	
		Control N(%)	Intervention N(%)	P-value	aOR (95% CI)**	Intra-class correlation (ICC)
**Patient satisfaction**						
Are you satisfied with services and would you recommend a friend?	No	9070 (42.1)	12694 (38.0)		Ref	
	Yes	12485 (57.9)	20670 (62.0)	0.00	1.66 (1.59-1.73)	0.275
**Patient experience**						
Were you adequately informed by the care provider about examinations, actions and decisions taken for your care?	No	5021 (23.4)	6437 (19.3)		Ref	
	Yes	16439 (76.6)	26882 (80.7)	0.00	0.55 (0.53-0.58)	0.507
Are you satisfied with education/information?	No	12227 (56.7)	16922 (50.7)		Ref	
	Yes	9328 (43.3)	16422 (49.3)	0.00	1.34 (1.29-1.40)	0.167

For peer review only - http://bmjopen.bmj.com/site/about/guidelines.xhtml

Are you satisfied with level of privacy?	No	10433 (48.4)	16350 (49.0)		Ref	
	Yes	11122 (51.6)	17014 (51.0)	0.167	1.14 (1.09-1.18)	0.136
Were you or your newborn physically, verbally or sexually abused during labor or childbirth or after birth?	No	21308 (98.9)	33149 (99.4)		Ref	
	Yes	247 (1.1)	215 (0.6)	0.00	0.42 (0.34-0.51)	0.082
Were you treated with respect and was your dignity preserved during your stay at the hospital?	No	275 (1.3)	290 (0.9)		Ref	
	Yes	21280 (98.7)	33074 (99.1)	0.00	1.81 (1.52-2.16)	0.063
Did you have a companion of your choice during labor and child birth?	No	17837 (83.1)	26991 (81.0)		Ref	
	Yes	3623 (16.9)	6328 (19.0)	0.00	0.27 (0.25-0.29)	0.687

*Binary logistic regression models adjusted for caste/ethnicity and education level

** GLMM models adjusted for clustering (by hospital) and caste/ethnicity and education level

For peer review only - http://bmjopen.bmj.com/site/about/guidelines.xhtml

For peer review only

For peer review only - http://bmjopen.bmj.com/site/about/guidelines.xhtml

Figure legends

Figure 1. WHO Framework for the Quality of maternal and newborn health care. Reprinted from the WHO document "Standards for improving quality of maternal and newborn care in health facilities", Page No. 16, Copyright World Health Organization (2016).

Figure 2. Stepped-wedge design and time line of the NePeriQIP trial. The period within bold lines represents the baseline period. The shaded period constitutes the intervention period. Each wedge contains three hospitals (clusters).

Figure 3. Participants flow diagram

For peer review only

For peer review only - http://bmjopen.bmj.com/site/about/guidelines.xhtml

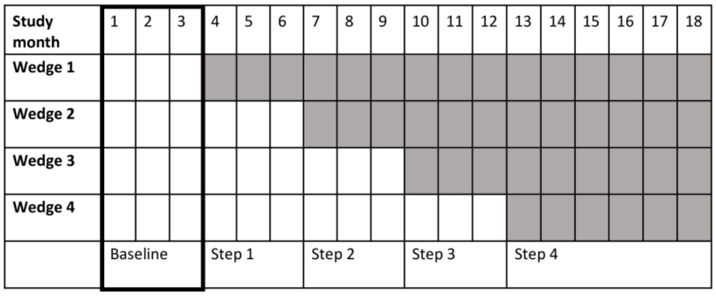

Figure 2. Stepped-wedge design and time line of the NePeriQIP trial. The period within bold lines represents the baseline period. The shaded period constitutes the intervention period. Each wedge contains three hospitals (clusters).

209x297mm (300 x 300 DPI)

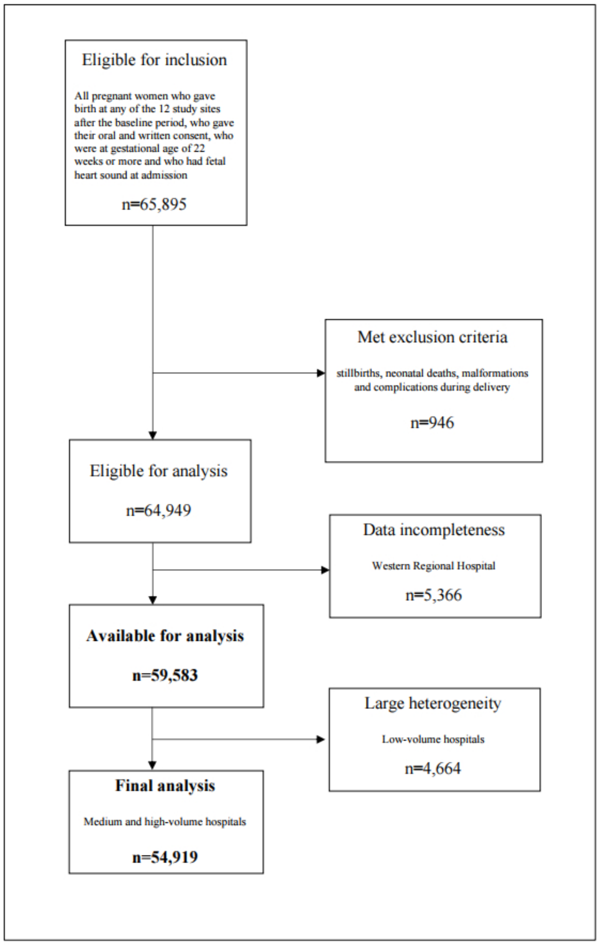

Figure 3. Participants flow diagram

209x296mm (96 x 96 DPI)

Table S1. Binary logistic regression of patient satisfaction and quality measures of experience of care, by hospital. Adjusted for caste/ethnicity and education level (n=54,919).

		Satisfaction			Informed			Educated			Privacy			Abuse			Respect			Companion		
**Hospital**		Yes N(%)	No N(%)	Adj OR (95% CI)	Yes N(%)	No N(%)	Adj OR (95% CI)	Yes N(%)	No N(%)	Adj OR (95% CI)	Yes N(%)	No N(%)	Adj OR (95% CI)	Yes N(%)	No N(%)	AdjOR (95% CI)	Yes N(%)	No N(%)	AdjOR (95% CI)	Yes N(%)	No N(%)	AdjOR (95% CI)
Mid Western Region	Control	194 (66.4)	98 (33.6)	Ref	284 (97.3)	8 (2.7)	Ref	27 (9.2)	265 (90.8)	Ref	20 (6.8)	272 (93.2)	Ref	1 (0.3)	291 (99.7)	Ref	290 (99.3)	2 (0.7)	Ref	2 (0.7)	290 (99.3)	Ref
	Intervention	2318 (54.1)	1966 (45.9)	0.59 (0.46-0.76)	4252 (99.3)	30 (0.7)	3.99 (1.81-8.80)	1525 (35.6)	2759 (64.4)	5.45 (3.65-8.14)	1171 (27.3)	3113 (72.7)	5.12 (3.24-8.11)	42 (1.0)	4242 (99.0)	2.81 (0.39-20.5)	4259 (99.4)	25 (0.6)	1.14 (0.27-4.84)	12 (0.3)	4270 (99.7)	0.38 (0.08-1.70)
Bharatpur	Control	751 (23.7)	2418 (76.3)	Ref	2945 (93.7)	198 (6.3)	Ref	863 (27.2)	2306 (72.8)	Ref	871 (27.5)	2298 (72.5)	Ref	102 (3.2)	3067 (96.8)	Ref	3057 (96.5)	112 (3.5)	Ref	1285 (40.9)	1858 (59.1)	Ref
	Intervention	4971 (52.3)	4527 (47.7)	3.57 (3.26-3.91)	9224 (97.2)	265 (2.8)	2.34 (1.94-2.83)	5708 (60.1)	3790 (39.9)	4.07 (3.72-4.45)	5091 (53.6)	4407 (46.4)	3.08 (2.82-3.37)	63 (0.7)	9435 (99.3)	0.20 (0.15-0.27)	9421 (99.2)	77 (0.8)	4.54 (3.39-6.09)	174 (1.8)	9315 (98.2)	0.03 (0.02-0.03)
Seti Zonal	Control	919 (41.3)	1304 (58.7)	Ref	1959 (88.2)	261 (11.8)	Ref	421 (18.9)	1802 (81.1)	Ref	867 (39.0)	1356 (61.0)	Ref	37 (1.7)	2186 (98.3)	Ref	2180 (98.1)	43 (1.9)	Ref	1623 (73.1)	597 (26.9)	Ref
	Intervention	3778 (58.9)	2636 (41.1)	2.07 (1.87-2.28)	6107 (95.3)	303 (4.7)	2.74 (2.30-3.26)	3056 (47.6)	3358 (52.4)	3.93 (3.50-4.42)	3658 (57.0)	2756 (43.0)	2.11 (1.92-2.33)	35 (0.5)	6379 (99.5)	0.32 (0.20-0.50)	6331 (98.7)	83 (1.3)	1.53 (1.05-2.21)	5814 (90.7)	596 (9.3)	3.59 (3.16-4.07)
Koshi Zonal	Control	2777 (85.6)	468 (14.4)	Ref	2190 (67.8)	1040 (32.2)	Ref	2327 (71.7)	918 (28.3)	Ref	2606 (80.3)	639 (19.7)	Ref	9 (0.3)	3236 (99.7)	Ref	3219 (99.2)	26 (0.8)	Ref	495 (15.3)	2735 (84.7)	Ref
	Intervention	3886 (90.7)	398 (9.3)	1.83 (1.57-2.12)	3148 (73.7)	1124 (26.3)	1.33 (1.20-1.47)	2841 (66.3)	1443 (33.7)	0.82 (0.74-0.90)	2859 (66.7)	1425 (33.3)	0.53 (0.48-0.60)	32 (0.7)	4252 (99.3)	2.59 (1.21-5.53)	4252 (99.3)	32 (0.7)	0.99 (0.58-1.69)	256 (6.0)	4016 (94.0)	0.36 (0.31-0.43)

For peer review only

Rapti Subregional	Control	785 (31.8)	1686 (68.2)	Ref	995 (40.3)	1473 (59.7)	Ref	569 (23.0)	1902 (77.0)	Ref	1108 (44.8)	1363 (55.2)	Ref	17 (0.7)	2454 (99.3)	Ref	2424 (98.1)	47 (1.9)	Ref	22 (0.9)	2446 (99.1)	Ref
	Intervention	271 (15.5)	1473 (84.5)	0.39 (0.34-0.46)	266 (15.3)	1478 (84.7)	0.27 (0.23-0.31)	96 (5.5)	1648 (94.5)	0.19 (0.15-0.24)	875 (50.2)	869 (49.8)	1.23 (1.09-1.39)	17 (1.0)	1727 (99.0)	1.42 (0.72-2.80)	1732 (99.3)	12 (0.7)	2.86 (1.51-5.42)	29 (1.7)	1715 (98.3)	1.97 (1.13-3.46)
Lumbini zonal	Control	5512 (87.5)	791 (12.5)	Ref	4445 (71.0)	1813 (29.0)	Ref	4032 (64.0)	2271 (36.0)	Ref	4378 (69.5)	1925 (30.5)	Ref	73 (1.2)	6230 (98.8)	Ref	6268 (99.4)	35 (0.6)	Ref	183 (2.9)	6075 (97.1)	Ref
	Intervention	4242 (88.3)	564 (11.7)	1.05 (0.94-1.18)	2739 (57.1)	2058 (42.9)	0.54 (0.50-0.58)	2830 (58.9)	1976 (41.1)	0.80 (0.74-0.87)	2987 (62.2)	1819 (37.8)	0.72 (0.66-0.78)	17 (0.4)	4789 (99.6)	0.31 (0.18-0.52)	4764 (99.1)	42 (0.9)	0.63 (0.40-0.98)	42 (0.9)	4755 (99.1)	0.30 (0.21-0.42)
Bheri Zonal	Control	1547 (40.2)	2305 (59.8)	Ref	3621 (94.1)	228 (5.9)	Ref	1089 (28.3)	2763 (71.7)	Ref	1272 (33.0)	2580 (67.0)	Ref	8 (0.2)	3844 (99.8)	Ref	3842 (99.7)	10 (0.3)	Ref	13 (0.3)	3836 (99.7)	Ref
	Intervention	1204 (51.6)	1130 (48.4)	1.62 (1.46-1.80)	1146 (49.3)	1179 (50.7)	0.61 (0.05-0.07)	386 (16.5)	1948 (83.5)	0.51 (0.45-0.58)	373 (16.0)	1961 (84.0)	0.39 (0.34-0.45)	9 (0.4)	2325 (99.6)	1.88 (0.72-4.87)	2315 (99.2)	19 (0.8)	0.32 (0.15-0.69)	1 (0.0)	2324 (100.0)	0.13 (0.02-0.96)
All	Control	12485 (57.9)	9070 (42.1)	Ref	16439 (76.6)	5021 (23.4)	Ref	9328 (43.3)	12227 (56.7)	Ref	11122 (51.6)	10433 (48.4)	Ref	247 (1.1)	21308 (98.9)	Ref	21280 (98.7)	275 (1.3)	Ref	3623 (16.9)	17837 (83.1)	Ref
	Intervention	20670 (62.0)	12694 (38.0)	1.20 (1.16-1.25)	26882 (80.7)	6437 (19.3)	1.26 (1.21-1.31)	16442 (49.3)	16922 (50.7)	1.28 (1.24-1.33)	17014 (51.0)	16350 (49.0)	0.98 (0.95-1.01)	215 (0.6)	33149 (99.4)	0.55 (0.46-0.66)	33074 (99.1)	290 (0.9)	1.49 (1.27-1.76)	6328 (19.0)	26991 (81.0)	1.12 (1.07-1.17)

For peer review only

For peer review only

CONSORT 2010 checklist of information to include when reporting a randomised trial*

Section/Topic	Item No	Checklist item	Reported on page No
**Title and abstract**			
	1a	Identification as a randomised trial in the title	1
	1b	Structured summary of trial design, methods, results, and conclusions (for specific guidance see CONSORT for abstracts)	2
**Introduction**			
Background and objectives	2a	Scientific background and explanation of rationale	4
	2b	Specific objectives or hypotheses	5
**Methods**			
Trial design	3a	Description of trial design (such as parallel, factorial) including allocation ratio	5
	3b	Important changes to methods after trial commencement (such as eligibility criteria), with reasons	N/A
Participants	4a	Eligibility criteria for participants	6
	4b	Settings and locations where the data were collected	5-6
Interventions	5	The interventions for each group with sufficient details to allow replication, including how and when they were actually administered	6
Outcomes	6a	Completely defined pre-specified primary and secondary outcome measures, including how and when they were assessed	7-9
	6b	Any changes to trial outcomes after the trial commenced, with reasons	N/A
Sample size	7a	How sample size was determined	9
	7b	When applicable, explanation of any interim analyses and stopping guidelines	N/A
Randomisation:			
Sequence generation	8a	Method used to generate the random allocation sequence	N/A
	8b	Type of randomisation; details of any restriction (such as blocking and block size)	N/A
Allocation concealment mechanism	9	Mechanism used to implement the random allocation sequence (such as sequentially numbered containers), describing any steps taken to conceal the sequence until interventions were assigned	N/A
Implementation	10	Who generated the random allocation sequence, who enrolled participants, and who assigned participants to interventions	N/A
Blinding	11a	If done, who was blinded after assignment to interventions (for example, participants, care providers, those	N/A

For peer review only - http://bmjopen.bmj.com/site/about/guidelines.xhtml

		assessing outcomes) and how	
	11b	If relevant, description of the similarity of interventions	N/A
Statistical methods	12a	Statistical methods used to compare groups for primary and secondary outcomes	9
	12b	Methods for additional analyses, such as subgroup analyses and adjusted analyses	9
**Results**			
Participant flow (a diagram is strongly recommended)	13a	For each group, the numbers of participants who were randomly assigned, received intended treatment, and were analysed for the primary outcome	10
	13b	For each group, losses and exclusions after randomisation, together with reasons	10
Recruitment	14a	Dates defining the periods of recruitment and follow-up	7
	14b	Why the trial ended or was stopped	N/A
Baseline data	15	A table showing baseline demographic and clinical characteristics for each group	17
Numbers analysed	16	For each group, number of participants (denominator) included in each analysis and whether the analysis was by original assigned groups	10-11
Outcomes and estimation	17a	For each primary and secondary outcome, results for each group, and the estimated effect size and its precision (such as 95% confidence interval)	10-11
	17b	For binary outcomes, presentation of both absolute and relative effect sizes is recommended	10-11
Ancillary analyses	18	Results of any other analyses performed, including subgroup analyses and adjusted analyses, distinguishing pre-specified from exploratory	10-11
Harms	19	All important harms or unintended effects in each group (for specific guidance see CONSORT for harms)	N/A
**Discussion**			
Limitations	20	Trial limitations, addressing sources of potential bias, imprecision, and, if relevant, multiplicity of analyses	13
Generalisability	21	Generalisability (external validity, applicability) of the trial findings	13
Interpretation	22	Interpretation consistent with results, balancing benefits and harms, and considering other relevant evidence	11-13
**Other information**			
Registration	23	Registration number and name of trial registry	14
Protocol	24	Where the full trial protocol can be accessed, if available	14
Funding	25	Sources of funding and other support (such as supply of drugs), role of funders	14

*We strongly recommend reading this statement in conjunction with the CONSORT 2010 Explanation and Elaboration for important clarifications on all the items. If relevant, we also recommend reading CONSORT extensions for cluster randomised trials, non-inferiority and equivalence trials, non-pharmacological treatments, herbal interventions, and pragmatic trials. Additional extensions are forthcoming: for those and for up to date references relevant to this checklist, see www.consort-statement.org.

For peer review only - http://bmjopen.bmj.com/site/about/guidelines.xhtml

BMJ Open

Effect of a perinatal care quality improvement package on patient satisfaction: a secondary outcome analysis of a cluster randomized control trial

Journal:	*BMJ Open*
Manuscript ID	bmjopen-2021-054544.R2
Article Type:	Original research
Date Submitted by the Author:	14-May-2022
Complete List of Authors:	Brunell, Olivia; Uppsala University, Department of Women's and Children's Health<br>Chaulagain, Dipak; Uppsala University, Department of Women's and Children's Health<br>KC, Ashish; Uppsala University, Department of Women's and Children's Health<br>Bergström, Anna; Uppsala University, Department of Women's and Children's Health<br>Målqvist, Mats; Uppsala University, Department of Women's and Children's Health
**Primary Subject Heading**:	Global health
Secondary Subject Heading:	Paediatrics
Keywords:	PAEDIATRICS, NEONATOLOGY, Quality in health care < HEALTH SERVICES ADMINISTRATION & MANAGEMENT

SCHOLARONE™
Manuscripts

I, the Submitting Author has the right to grant and does grant on behalf of all authors of the Work (as defined in the below author licence), an exclusive licence and/or a non-exclusive licence for contributions from authors who are: i) UK Crown employees; ii) where BMJ has agreed a CC-BY licence shall apply, and/or iii) in accordance with the terms applicable for US Federal Government officers or employees acting as part of their official duties; on a worldwide, perpetual, irrevocable, royalty-free basis to BMJ Publishing Group Ltd ("BMJ") its licensees and where the relevant Journal is co-owned by BMJ to the co-owners of the Journal, to publish the Work in this journal and any other BMJ products and to exploit all rights, as set out in our licence.

The Submitting Author accepts and understands that any supply made under these terms is made by BMJ to the Submitting Author unless you are acting as an employee on behalf of your employer or a postgraduate student of an affiliated institution which is paying any applicable article publishing charge ("APC") for Open Access articles. Where the Submitting Author wishes to make the Work available on an Open Access basis (and intends to pay the relevant APC), the terms of reuse of such Open Access shall be governed by a Creative Commons licence – details of these licences and which Creative Commons licence will apply to this Work are set out in our licence referred to above.

Other than as permitted in any relevant BMJ Author's Self Archiving Policies, I confirm this Work has not been accepted for publication elsewhere, is not being considered for publication elsewhere and does not duplicate material already published. I confirm all authors consent to publication of this Work and authorise the granting of this licence.

For peer review only

Effect of a perinatal care quality improvement package on patient satisfaction: a secondary outcome analysis of a cluster randomized control trial

Olivia Brunell[1], Dipak Chaulagain[1], Ashish KC[1], Anna Bergström[1], Mats Målqvist[1]

[1] Department of Women's and Children's Health, Uppsala University

Correspondence to:

Olivia Brunell, Department of Women's and Children's Health, Uppsala University,

Sjukhusvägen 7, 753 09 Uppsala, Sweden

e-mail: olivia.brunell@kbh.uu.se

phone: +46 8611 00 00

Keywords

Neonatology, Perinatal care, Quality Improvement, Patient Satisfaction

Word count 3546

ABSTRACT

For peer review only

Objective To investigate the effect of a quality improvement (QI) package on patient satisfaction of perinatal care.

Design Secondary analysis of a stepped-wedge cluster randomized controlled trial. Participating hospitals were randomized by size into four different wedges.

Setting 12 secondary-level public hospitals in Nepal.

Participants Women who gave birth in the hospitals at a gestational age of ≥22weeks, with fetal heart sound at admission. Adverse outcomes were excluded. One hospital was excluded due to data incompleteness and four low-volume hospitals due to large heterogeneity. The final analysis included 54,919 women.

Intervention Hospital management was engaged and facilitators were recruited from within hospitals. Available perinatal care was assessed in each hospital, followed by a bottle-neck analysis workshop. A three-day training in essential newborn care was carried out for health workers involved in perinatal care, and a set of quality improvement tools were introduced to be used in every day practice (skill-checks, self- assessment checklists, scoreboards, and weekly Plan-Do-Study-Act-meetings). Refresher training after 6 months.

Outcome measure Women's satisfaction with care during childbirth (a prespecified secondary outcome).

Results The likelihood of women being overall satisfied with care during childbirth increased after the intervention, [aOR 1.66(1.59-1.73)]. However, the proportions of overall satisfaction were low (control 58%, intervention 62%). Women were more likely to be satisfied with education and information from health workers after intervention [aOR 1.34(1.29-1.40)] and to have been treated with dignity and respect [aOR 1.81(1.52-2.16)]. The likelihood of having experienced abuse during the hospital stay decreased [aOR: 0.42(0.34-.051)] and of being satisfied with the level of privacy increased [aOR 1.14, 95%(1.09-1.18)].

For peer review only - http://bmjopen.bmj.com/site/about/guidelines.xhtml

Conclusions Improvements in patient satisfaction were indicated after the introduction of a QI-package on perinatal care. We recommend further studies on which aspects of care are most important to improve women's satisfaction of perinatal care in hospitals in Nepal.

Trial registration number ISRCTN30829654.

ARTICLE SUMMARY

Strengths and limitations of this study

- This secondary analysis focuses on patients' experience of care, an important dimension of quality improvement which is often overlooked when evaluating quality improvement efforts.
- The large sample size from different hospitals increases representativeness of the sample and generalizability of our results, while external validity is strengthened by the use of the WHO framework.
- The stepped-wedge cluster-randomization allowed us to eventually roll out the intervention at all sites, while mimicking a randomized-controlled trial.
- The exclusion of one high-volume hospital and the four low- volume hospitals, deviates from the original stepped-wedged design and should be considered when interpreting the results.
- We measured the WHO domains of experience of care and no other factors that are also known to affect satisfaction, such as waiting times, overcrowding and physical resources in the health facility.

For peer review only

INTRODUCTION

In 2017 an estimated 2,5 million children died in the neonatal period whereof about 1 million newborn died during their first day of life[1]. Though an increasing number of women are giving birth in health facilities it has not ensured improved health care outcomes[2]. The quality of perinatal care provided in health facilities needs improvement to end preventable mortality[2-4]. Both Quality of Care (QoC) and approaches to its assessment are complex [5]. The definition of QoC by the World Health Organization (WHO) is "The extent to which health care services provided to individuals and patient populations improve desired health outcomes" and to achieve this, health care must be safe, effective, timely, efficient, equitable and people-centered. Thus, health care must not only provide evidence based clinical care, but by being people-centered, the care must also be provided considering individuals preferences, needs and values and the culture of their communities[6]. This is important as patients have the right to be treated with dignity and respect, but also because people-centered care is associated with improved health care utilization and health outcomes[7]. In 2016, WHO developed a framework and standards with accompanying quality measures for the quality of maternal and newborn health care. In this framework, QoC is divided into two interlinked dimensions; (1) *Provision of clinical care* by health workers, and (2) patients' *Experience of care*[8]. *Experience of care* is a people-centered measure, a process indicator which reflects the interactions that patients have with the healthcare system[9]. It is composed of three major domains; effective communication, respect and dignity, and emotional support[6]. Patient satisfaction, together with other people-centered outcomes, is an outcome of *Experience of care*. [9-11].

In 2013 the research group conducted a study of the implementation of a quality improvement (QI) package for neonatal resuscitation in a tertiary hospital in Nepal. In this study the

For peer review only - http://bmjopen.bmj.com/site/about/guidelines.xhtml

Helping Babies Breathe (HBB) program, developed by The American Academy of Pediatrics to improve health workers' performance on basic resuscitation[12, 13], was implemented. In addition to HBB-training, the intervention included QI-components, aiming to continuously reinforce the HBB protocol throughout the intervention period and to motivate both hospital management and individual health workers to be engaged in improvement.

The study, Helping Babies Breath-Quality Improvement Cycle (HBB-QIC), showed improvement in health workers adherence to neonatal resuscitation protocols and a large decrease in intrapartum still births and first-day neonatal mortality [14]. These positive results are more pronounced than in previous studies of implementation of the HBB-program [15, 16] and can be due to the addition of the QI components.

However, there were no significant change of overall in-hospital perinatal mortality, which indicates that efforts are also needed to improve the continued postnatal care[17]. Based on the findings from HBB-QIC, a scale-up was developed by the research group in collaboration with Ministry of Health and Population in Nepal. This is called Nepal Perinatal Quality Improvement Project (NePeriQIP) and aims to improve quality of perinatal care through increased clinical skills and knowledge in essential newborn care (ENC) and establish a structure for continuous quality improvement[18]. The primary objective of the NePeriQIP trial was to evaluate impact on intrapartum mortality (intrapartum stillbirth and first-day mortality), and the results showed a significant reduction in intrapartum-related deaths (intrapartum stillbirths and first day mortality) in the intervention period (aOR, 0.79; 95% CI, 0.69–0.92)[19]. In the present manuscript, we aim to evaluate the effect of NePeriQIP on the prespecified secondary outcome *patient satisfaction*.

METHODS

Trial design

For peer review only - http://bmjopen.bmj.com/site/about/guidelines.xhtml

A stepped-wedge cluster-randomized design was used[18], where 12 hospitals were randomized by size into four wedges. Each wedge included one high-volume (>8000 deliveries a year), one medium-volume (>3000 deliveries a year) and one low-volume (>1000 deliveries a year) hospital. Each cluster had a control and an intervention period. During the first three months of the study period no intervention activities took place at any of the hospitals (baseline period). NePeriQIP was then introduced to one wedge with three hospitals at a time, with a three months delay between wedges, and eventually all hospitals were exposed to the intervention (Figure 1).

Setting

The selected 12 hospitals filled the criteria of having >1000 deliveries per year and being a referral center for maternal and newborn care. Although most of the hospitals were located in the flatlands, they were diverse in relation to ethnicity, language and religion. All hospitals provided vaginal and cesarian deliveries, and had access to neonatal resuscitation services at birth. Skilled birth-attendants (obstetricians, medical doctors, senior nurses or senior nurse midwives) led the labor units. Medical doctors led the pediatric units where sick newborns were managed in the low-volume hospitals. Pediatricians led the specialized sick newborn care units in the medium and high-volume hospitals. The intrapartum related mortality rate (intrapartum stillbirth and first day neonatal mortality) ranged from 9 to 31 per 1,000 births at the hospitals (mean 13/1000 births), during the baseline period[19].

Participants

All pregnant women who gave birth at any of the 12 hospitals after the baseline period, who gave their oral and written consent, who were at gestational age of 22 weeks or more and who had fetal heart sound at admission were eligible for inclusion. Women who experienced

For peer review only

stillbirths, neonatal deaths, malformations and complications during childbirth were excluded as these events could bias patient satisfaction.

The intervention

The development of the QI-package was guided by the integrated-Promoting Action on Research Implementation in Health Services (iPARIHS) framework [20]. Ministry of Health, together with the research team, developed an implementation guideline for the implementation process. In each hospital, the QI-package was introduced in the following steps: first, the hospital management was oriented on the QI-package. They appointed in-hospital facilitators from among pediatricians, medical officers and nurses to facilitate the implementation, supported by the study team. The number of facilitators depended on the size of the hospital: two from low-volume, three from medium-volume and four from high-volume-hospitals. The facilitators participated in a seven-day training on the QI-package, provided by the study team. Following the training, the QI facilitators assessed service readiness and availability in their respective hospital, using a checklist developed by the team, and a bottleneck analysis workshop was organized based on that. After these initial steps, a three-day on-site training for all health staff involved in perinatal care was organized by the facilitators and the study team. The training consisted of education and skills-training in ENC and introduction of a set of QI-tools. The ENC component included resuscitation and immediate newborn care (thorough drying, skin-to-skin contact, delayed clamping of the cord and early initiation of breastfeeding) and were taught using the HBB training manual version one[12]. In addition, lectures were given on breastfeeding, infection prevention and kangaroo mother care for low birth-wight newborns. The QI- tools included daily bag-and-mask ventilation skill check on an HBB-mannequin (the low-cost simulator Laerdal® NeoNatalie), checklist for preparations before each birth, self-assessment checklist of performed

For peer review only - http://bmjopen.bmj.com/site/about/guidelines.xhtml

resuscitation, and use of score-boards comprising major indicators on neonatal resuscitation. In addition to these tools a structured model for quality improvement, Plan-Do-Study-Act (PDSA)-cycle , was used aiming to identify and act upon local problems [21]. After the training, the QI facilitators initiated weekly PDSA-meetings as part of routine work at all units, involving health workers related to perinatal care. The duration of each meeting was about half an hour. A one-day refresher training in HBB was held for health workers after six months.

Conceptual framework

The WHO conceptual framework on QoC[6] and the WHO-document "Standards for improving quality of maternal and newborn care in health facilities"[8] were used to guide data collection and analyzes for this manuscript. In the framework, QoC consists of the two inter-linked dimensions 'Provision of care' and 'Experience of care'. Experience of care contains three domains; *effective communication*, *preservation of respect and dignity*, and *emotional support*. Though NePeriQIP focus on clinical provision of care, a successful implementation where health workers are more engaged in the quality of perinatal care could generate better experiences among patients and manifest in patient satisfaction.

Data collection and variables

The study period followed the Nepali calendar and was initiated on June 2017 and ran for 15 months up until October 2018. Data for this manuscript was collected through structured interviews with all included mothers upon discharge, throughout the study period. An independent data collection team was established at each hospital and a structured questionnaire developed by the study team and piloted on beforehand in a hospital later not included in the study, was used to collect the data. After completion of the data collection

For peer review only - http://bmjopen.bmj.com/site/about/guidelines.xhtml

forms, they were sent weekly to the central research office in Kathmandu, where they were entered into a Census and Survey Processing System database by a team of independent data entry officers.

The collected data from exit interviews was not accessible for the local teams during the intervention. Results will be shared with the local teams by the published paper.

In this manuscript, we report the findings from the trial on patient satisfaction (a prespecified secondary outcome). This was measured through *Overall satisfaction* and the three dimensions of *Experience of care* (effective communication, respect and dignity, and emotional support) in the WHO Quality of Care framework for maternal and newborn health [6].

Overall satisfaction was measured as an aggregate score of two questions, and to be considered satisfied with services received, mothers needed to respond favorably to both questions.

(1) *Overall, how satisfied are you with the services?*

(Recorded on a Likert scale of 1 to 5. 1= very dissatisfied, 2= dissatisfied, 3= neither, 4= satisfied, 5= very satisfied. Scores were dichotomized, with an answer of 4 or more being set as satisfied).

(2) *Would you recommend a friend to give birth at this hospital?*

(recorded yes/ no)

Experience of care was investigated through a set of questions suggested as quality measures of experience of care in the WHO-document "Standards for improving quality of maternal and newborn care in health facilities"[8].

For peer review only

(1) Patient communication

Were you adequately informed by the health care worker about examinations, actions and decisions taken for your care throughout the hospital stay?

(recorded yes/no)

Are you satisfied with health education and information received from health care providers?

(Recorded on a Likert scale of 1 to 5. 1= very dissatisfied, 2= dissatisfied, 3= neither, 4= satisfied, 5= very satisfied. Scores were dichotomized, with an answer of 4 or more being set as satisfied).

(2) Respect and dignity

Are you satisfied with the degree of privacy during your stay in labor and child birth areas?

(Recorded on a Likert scale of 1 to 5. 1= very dissatisfied, 2= dissatisfied, 3= neither, 4= satisfied, 5= very satisfied. Scores were dichotomized, with an answer of 4 or more being set as satisfied).

Where you or your newborn physically, verbally or sexually abused during labor or childbirth or after birth?

(recorded yes/no)

Were you treated with respect and was your dignity preserved during your stay at the hospital?

(recorded yes/no)

(3) Emotional support

Did you have a companion of your choice during labor and childbirth?

For peer review only - http://bmjopen.bmj.com/site/about/guidelines.xhtml

(recorded yes/no)

Statistical methods

There was no a priori estimation of sample size for this secondary outcome analysis, it was calculated for the primary outcome of the NePeriQIP-trial, intrapartum mortality[19] . Given that intrapartum mortality is a rare outcome, this rendered a large sample-size, which allowed for the detection of small differences in the outcome of interest for the present manuscript. Pearson's Chi-Squared test was used to analyze background characteristics of participants in the control and intervention groups, performed with Statistical Package for Social Sciences (SPSS) version 25.0. Change in satisfaction and experiences of care between the control and intervention groups was analyzed by generalized linear mixed models (GLMM) using the software R (version 3.4.0). Analyses were performed with R package lme4. Adjustments were made for structural factors (cast/ethnicity and educational level) as these could be ground for discrimination and altered treatment. Intra-cluster correlations coefficients (ICC) for each outcome were calculated. Initial analyses showed high ICC for all outcome variables, making the results inconclusive. Further analysis of the data by hospital displayed that the low-volume hospitals showed large heterogeneity. As a consequence, the final GLMM analysis was performed on the medium and high-volume hospitals. Analysis of change before and after intervention at each hospital was performed with binary logistic regression of satisfaction and experience of care (adjusted for caste/ethnicity and education level) with Statistical Package for Social Sciences (SPSS) version 25.0. Significance level of 95% was used.

Ethical considerations

Ethical approval was obtained from the Nepal Health Research Council (ref 26–2017). The data used for this secondary outcome analysis was collected through face-to-face interviews

For peer review only

after obtaining written informed consent from the participants. The identity of the participants was coded after data collection, and the code key was stored in a safe environment that only the project management had access to.

Patient and public involvement

Patients were not directly involved in developing the trial; however, it was planned and conducted in collaboration with Ministry of Health in Nepal.

RESULTS

A total number of 65,895 women were eligible for inclusion, whereof 946 women were excluded based on exclusion criteria. Due to data incompleteness from one of the hospitals, another 5,366 women were excluded, leaving 59,583 women available for analysis. Initial analysis revealed that low-volume hospitals showed large heterogeneity and differed from the medium and high-volume hospitals. As a consequence of this finding, the final GLMM analysis was performed on the medium and high-volume hospitals where 92% of deliveries took place, n= 54,919 (Figure 2).

Characteristics

A majority of the women had an education higher than primary (69%) and belonged to an advantageous cast (67%). About half of the women were first-time mothers (51%) but few were adolescents (7.6%) and the coverage of antenatal care was high (80% had four antenatal care visits before giving birth). The cesarean section rate was 19% and most of them (73%) were emergency cesarean sections. The proportion of preterm births were 13% and babies with low birth weight were 14% (Table 1).

For peer review only

For peer review only - http://bmjopen.bmj.com/site/about/guidelines.xhtml

Outcomes

Women were more likely to be satisfied with care after the intervention than before, adjusted Odds Ratio (aOR) 1.66 [CI: 1.59-1.73, ICC: 0.275]). The results varied between hospitals (aOR 0.39-3.57) (Table S1) and the overall proportion of satisfaction was low. Prior to the intervention a proportion of 58% of the women were satisfied, which increased to 62% after (Table 2).

The domains of experience of care were affected in a positive direction by the intervention. Women were more likely to be *satisfied with education and information* from health workers after implementation (aOR 1.34 [CI: 1.29-1.40], ICC: 0.167) as well as more likely to state that they had been *treated with preserved dignity and respect* (aOR: 1.81 [CI: 1.52-2.16], ICC: 0.063]). The likelihood of *Experience abuse during the hospital stay* decreased after the implementation (aOR: 0.42 [CI: 0.34-.051, ICC: 0.082]) and the likelihood of being *satisfied with the level of privacy* increased (aOR 1.14 [CI: 1.09-1.18, ICC: 0.136]) (Table 2). Two measures seemed to have been affected in a negative direction after the intervention: the likelihood of being *adequately informed about examinations, actions and decisions taken for their care* (aOR 0.55 [CI: 0.53-0.58, ICC: 0.507] and *having a companion of choice during labor* (aOR 0.27 [CI:0.25-0.29, ICC: 0.687]. These two measures, however, displayed relatively high ICC in the GLMM model, rendering the adjusted odds ratios to be inconclusive. (Table 2).

A very high proportion of women stated that they were treated with dignity and respect (before intervention 98.7%, after 99.1%) and very few had experienced abuse during their hospital stay (1.1% before intervention and 0.6% after). The proportion of women who stated that they were adequately informed about their care were 76.3% before intervention and

For peer review only

80.6% after. However, not many women had a companion of choice during labor (16.8% before and 19.0% after), and the proportions of women who were satisfied with the information and education from health workers, and with the level of privacy, were also low (43.3% before and 49.3% after, 51.6% before and 51.0% after, respectively) (Table 2).

DISCUSSION

The quality of perinatal care in health facilities needs to be improved to save newborn lives [2-4]. Measuring patient satisfaction is important in several aspects to improve quality of care; It can reveal gaps in the health system to address, it creates accountability among health care providers and stakeholders, it is useful to guide and evaluate QI efforts, and in addition satisfaction is known to affects patients care-seeking behavior and compliance[9, 22-24]. Our analysis shows that after the introduction of the QI-package of NePeriQIP, the likelihood that women were satisfied with perinatal care increased. Satisfaction is an outcome measure of experience of care [9], and in our analysis the people-centered quality measures of experience of care suggested by WHO were affected in a positive direction, along with satisfaction. The exact mechanisms behind our results are not clear as the intervention did not specifically focus on people-centered skills. However, besides that NePeriQIP focused on improvement of provision of care through training, it also included QI-tools to strengthen professional attitudes among health workers and sustain change in practice. Self-evaluation, insight in the ongoing progress and involvement in problem-solving promote a higher level of learning which can create a deeper understanding among health workers and make them pay more attention to what they are doing and why [25]. This can improve their capacity to inform and educate patients, to handle stressful situations and provide more time and engagement to attend to interpersonal interactions with patients. With increased knowledge and continuous

For peer review only - http://bmjopen.bmj.com/site/about/guidelines.xhtml

discussions concerning care of the mothers and newborn, healthcare workers might also get more sensitized and empathic towards their patients. A recent implementation study conducted in India, also adopting the PDSA-approach and facilitation to improve quality of care, could similarly to our results show improvements in satisfaction among women who had recently given birth [26].

Satisfaction is a complex phenomenon influenced by several determinants, and we cannot conclude that the improvement in satisfaction is explained solely by improvement in the measures of experience of care that we have studied. Other known determinants of satisfaction are structural elements (i.e., cleanliness of the health facility and availability of human recourses, medicines and supplies) and outcome related determinants such as health status of the mother and newborn[27]. Nevertheless, it is previously shown that the inter-personal components of experience of care dominate satisfaction with maternity care in Nepal and other low-income countries[27, 28].

Though we could see an increased satisfaction after the intervention, the proportion of satisfied women both before and after was low. This despite that women tend to report more satisfied with care if interviewed early after childbirth, biased by social desirability and the joy of having a healthy baby [29] and previous studies of satisfaction in Nepal and India has shown higher levels [26, 30, 31]. We do not know why the women were not more satisfied with care but some factors can be considered. First, we sought to reduce the influence of social desirability by our definition of satisfaction (overall satisfaction combined with whether to recommend the facility to a friend). In addition, other determinants that was not the scope of our analysis, could be of importance. Other studies of satisfaction with perinatal care in Nepal has shown that having a protected waiting area and having an opportunity to ask

For peer review only - http://bmjopen.bmj.com/site/about/guidelines.xhtml

questions are positively associated with patient satisfaction, while over-crowding and long waiting times has been shown to increase the likelihood of dissatisfaction[28, 31].

We adjusted for the structural determinants cast/ethnicity and educational level, which can be grounds for discrimination, altered treatment and outcomes. The intermediate determinants displayed in Table 1 were not considered grounds for discrimination and thus not included in the further analysis. Given the complexity of the stepped-wedge design, no further multi-level analyses were performed

The main strengths of our secondary outcome analysis are the large sample and the stepped-wedge cluster-randomization. The latter allowed us to eventually roll out the intervention at all sites, while mimicking a randomized-controlled trial. It was also guided by WHO framework which strengthens external validity.

There are some limitations worth mentioning. We excluded women with adverse outcomes since this could over shadow the experience of care and skew our results. We had to exclude one high-volume hospital due to data incompleteness and the four low-volume hospitals due to initial analysis showing high ICC. This deviates from the original stepped-wedged design and should be considered when interpreting the results. We only focused on measuring the WHO domains of experience of care and no other factors that are also known to affect satisfaction, such as waiting times, overcrowding, patients' expectations, provision of medical care or physical resources in the health facility[28, 32]. A more in-depth understanding of the women's satisfaction could have been achieved by adding qualitative interviews.

CONCLUSION

Measuring patient satisfaction is an important aspect of quality-of-care development and evaluation. We found that after the introduction of a QI-package (NePeriQIP), women giving

For peer review only - http://bmjopen.bmj.com/site/about/guidelines.xhtml

For peer review only

birth in health facilities were significantly more likely to be satisfied with care. Given the large sample size and the modest increase in the proportion of overall stated satisfaction, the results should be interpreted with caution. As satisfaction with perinatal care in Nepal still needs improvement, we recommend further studies on which aspects of care that are of most importance to improve patient satisfaction.

ACKNOWLEDGMENTS

The authors would like to thank the management and health care staff of all participating hospitals as well as facilitators, mentors, data collectors, and the data management team at Golden Community. Thanks to Abhishek Gurung, Elisha Joshi, Sunil Gajurel, Prajwal Poudel and Asmita Paudel for facilitating the QI implementation process in hospitals. Special thanks to Omkar Basnet for excellent work in data management and to Regina Gurung for participating in managing the project. Lastly, we would like to express our gratitude towards all participating mothers.

Contributors OB, MM, AB and AK conceptualized the study. AK and DC supervised data collection and management. OB and MM analyzed data and prepared the first draft, as well as the final version. All authors, reviewed, commented and approved the final version of the manuscript.

Trial registration number ISRCTN30829654.

Funding This work was supported by Swedish Research Council (2016-05621), the Laerdal Foundation for Acute Medicine, Norway (40198), and Einhorn Family Foundation, Sweden.

For peer review only - http://bmjopen.bmj.com/site/about/guidelines.xhtml

The funders had no role in study design, data collection and analysis, decision to publish, or preparation of the manuscript.

Competing interests There are no competing interests for any author.

Data availability statement Deidentified participant data used in the analysis of this manuscript will be available upon request. The study protocol has been published [18].

For peer review only

REFERENCES

1. Hug L, Sharrow D, Zhong K, You D: **Levels & Trends in Child Mortality**. In.: United Nations Inter-agency Group for Child Mortality Estimation (UN IGME); 2018.
2. Scott KW, Jha AK: **Putting quality on the global health agenda**. *N Engl J Med* 2014, **371**(1):3-5.
3. Kruk M, Gage A, Joseph N, Danaei G, Garcia-Saisó S, Salomon JA: **Mortality due to low-quality health systems in the universal health coverage era: a systematic analysis of amenable deaths in 137 countries**. *The Lancet* 2018, **392**(10160):2203-2212.
4. Bhutta Z, Das JK, Bahl R, Lawn JE, Salam RA, Vinod K Paul VK, Sankar MJ, Blencowe H, Rizvi A, Chou VB *et al*: **Every Newborn: Can available interventions end preventable deaths in mothers, newborn babies, and stillbirths, and at what cost?** . *The Lancet* 2014.
5. Hanefeld J, Powell-Jackson T, Balabanova D: **Understanding and measuring quality of care: dealing with complexity**. *Bull World Health Organ* 2017, **95**(5):368-374.
6. Tuncalp, Were WM, MacLennan C, Oladapo OT, Gulmezoglu AM, Bahl R, Daelmans B, Mathai M, Say L, Kristensen F *et al*: **Quality of care for pregnant women and newborns-the WHO vision**. *BJOG* 2015, **122**(8):1045-1049.
7. Doyle C, Lennox L, Bell D: **A systematic review of evidence on the links between patient experience and clinical safety and effectiveness**. *BMJ Open* 2013, **3**(1).
8. WHO.: **Standards for improving quality of maternal and newborn care in health facilities**. In. Geneva: WHO Press; 2016.
9. Larson E, Sharma J, Bohren MA, Tuncalp O: **When the patient is the expert: measuring patient experience and satisfaction with care**. *Bull World Health Organ* 2019, **97**(8):563-569.
10. Donabedian A: **The Definition of Quality and Approaches to Its Assessment, vol 1: Explorations in Quality Assessment and Monitoring**. University of Michigan: Health Administration Press; 1980.
11. Cleary PD, McNeil BJ: **Patient satisfaction as an indicator of quality care**. *Inquiry* 1988, **25**(1):25-36.

For peer review only - http://bmjopen.bmj.com/site/about/guidelines.xhtml

12. Singhal N, Lockyer J, Fidler H, Keenan W, Little G, Bucher S, Qadir M, Niermeyer S: **Helping Babies Breathe: global neonatal resuscitation program development and formative educational evaluation**. *Resuscitation* 2012, **83**(1):90-96.
13. Niermeyer S: **From the Neonatal Resuscitation Program to Helping Babies Breathe: Global impact of educational programs in neonatal resuscitation**. *Semin Fetal Neonatal Med* 2015, **20**(5):300-308.
14. Kc A, Wrammert J, Clark RB, Ewald U, Vitrakoti R, Chaudhary P, Pun A, Raaijmakers H, Malqvist M: **Reducing Perinatal Mortality in Nepal Using Helping Babies Breathe**. *Pediatrics* 2016, **137**(6).
15. Msemo G, Massawe A, Mmbando D, Rusibamayila N, Manji K, Kidanto HL, Mwizamuholya D, Ringia P, Ersdal HL, Perlman J: **Newborn mortality and fresh stillbirth rates in Tanzania after helping babies breathe training**. *Pediatrics* 2013, **131**(2):e353-360.
16. Goudar SS, Somannavar MS, Clark R, Lockyer JM, Revankar AP, Fidler HM, Sloan NL, Niermeyer S, Keenan WJ, Singhal N: **Stillbirth and newborn mortality in India after helping babies breathe training**. *Pediatrics* 2013, **131**(2):e344-352.
17. Wrammert J, Kc A, Ewald U, Malqvist M: **Improved postnatal care is needed to maintain gains in neonatal survival after the implementation of the Helping Babies Breathe initiative**. *Acta Paediatr* 2017, **106**(8):1280-1285.
18. Kc A, Bergstrom A, Chaulagain D, Brunell O, Ewald U, Gurung A, Eriksson L, Litorp H, Wrammert J, Gronqvist E *et al*: **Scaling up quality improvement intervention for perinatal care in Nepal (NePeriQIP); study protocol of a cluster randomised trial**. *BMJ Glob Health* 2017, **2**(3):e000497.
19. Kc A, Ewald U, Basnet O, Gurung A, Pyakuryal SN, Jha BK, Bergstrom A, Eriksson L, Paudel P, Karki S *et al*: **Effect of a scaled-up neonatal resuscitation quality improvement package on intrapartum-related mortality in Nepal: A stepped-wedge cluster randomized controlled trial**. *PLoS Med* 2019, **16**(9):e1002900.
20. Harvey G, Kitson A: **PARIHS revisited: from heuristic to integrated framework for the successful implementation of knowledge into practice**. *Implement Sci* 2016, **11**:33.
21. Christoff P: **Running PDSA cycles**. *Curr Probl Pediatr Adolesc Health Care* 2018, **48**(8):198-201.
22. Al-Abri R, Al-Balushi A: **Patient satisfaction survey as a tool towards quality improvement**. *Oman Med J* 2014, **29**(1):3-7.
23. Quintana JM, Gonzalez N, Bilbao A, Aizpuru F, Escobar A, Esteban C, San-Sebastian JA, de-la-Sierra E, Thompson A: **Predictors of patient satisfaction with hospital health care**. *BMC Health Serv Res* 2006, **6**:102.
24. Bernhart MH, Wiadnyana IG, Wihardjo H, Pohan I: **Patient satisfaction in developing countries**. *Soc Sci Med* 1999, **48**(8):989-996.
25. Anderson L.W. KDR, Bloom B.S: **A Taxonomy for Learning, Teaching, and Assessing : a Revision of Bloom's Taxonomy of Educational Objectives / Editors, Lorin W. Anderson, David Krathwohl ; Contributors, Peter W. Airasian ... [et Al.]**. . New York: Longman; 2001.
26. Das MK, Arora NK, Dalpath SK, Kumar S, Kumar AP, Khanna A, Bhatnagar A, Bahl R, Nisar YB, Qazi SA *et al*: **Improving quality of care for pregnancy, perinatal and newborn care at district and sub-district public health facilities in three districts of Haryana, India: An Implementation study**. *PLoS One* 2021, **16**(7):e0254781.

For peer review only

27. Srivastava A, Avan BI, Rajbangshi P, Bhattacharyya S: **Determinants of women's satisfaction with maternal health care: a review of literature from developing countries**. *BMC Pregnancy Childbirth* 2015, **15**:97.
28. Mehata S, Paudel YR, Dariang M, Aryal KK, Paudel S, Mehta R, King S, Barnett S: **Factors determining satisfaction among facility-based maternity clients in Nepal**. *BMC Pregnancy Childbirth* 2017, **17**(1):319.
29. Lumley J: **Assessing satisfaction with childbirth**. *Birth* 1985, **12**(3):141-145.
30. Ministry of Health and Population (MOHP) [Nepal] HRaSDFH, and Nepal Health Sector Support Programme (NHSSP): **Service Tracking Survey 2013**. In. Kathmandu, Nepal: Ministry of Health and Population, HERD, and NHSSP, Ramsah Path, Kathmandu, Nepal; 2014.
31. Acharya S, S. Sharma, B. Dulal, K. Aryal: **Quality of Care and Client Satisfaction with Maternal Health Services in Nepal: Further Analysis of the 2015 Nepal Health Facility Survey. DHS Further Analysis Reports No. 112**. In. Rockville, Maryland, USA: ICF; 2018.
32. Bjertnaes OA, Sjetne IS, Iversen HH: **Overall patient satisfaction with hospitals: effects of patient-reported experiences and fulfilment of expectations**. *BMJ Qual Saf* 2012, **21**(1):39-46.

TABLES

Table 1. Characteristics of study participants in control and intervention group

Chi2-test to detect group differences before and after NePeriQIP intervention start.

n_{tot}=59,583.

**Demographic characteristics**		**Total** n (%)	**Control** n (%)	**Intervention** n (%)	**p-value**
Structural determinants					
Mother's cast/ethnicity	relatively disadvantaged	19,667 (33.0)	8,537 (35.9)	11,130 (31.1)	<0.001*
	relatively advantaged	39,916 (67.0)	15,270 (64.1)	24,646 (68.9)	
Mother's education	Primary or less	18,591 (31.3)	7,572 (31.9)	11,019 (30.9)	0.006*
	Secondary or higher	40,829 (68.7)	16,139 (68.1)	24,690 (69.1)	
Intermediate determinants					

For peer review only - http://bmjopen.bmj.com/site/about/guidelines.xhtml

Adolescent mother (≤19 years)	No	55,061 (92.4)	22,026 (92.5)	33,035 (92.3)	0.415
	Yes	4,522 (7.6)	1,781 (7.5)	2,741 (7.7)	
Elder mother (>35 years)	No	59,012 (98.9)	23,667 (98.9)	35,345 (98.8)	0.082
	Yes	681 (1.1)	250 (1.1)	431 (1.2)	
Parity	Nulli	30,610 (51.4)	12,400 (52.1)	18,210 (50.9)	0.005*
	Multi	28,973 (48.6)	11,407 (47.9)	17,566 (49.1)	
>Four ANC visits	No	11,700 (19.9)	5,136 (21.9)	6,564 (18.5)	0.000*
	Yes	47,131 (80.1)	18,273 (78.1)	28,858 (81.5)	
Spontaneous vaginal	No	14,628 (23.3)	5,294 (22.2)	8,704 (24.3)	<0.001*
	Yes	45,585 (76.7)	18,513 (77.8)	27,072 (75.7)	
Elective CS	No	56,563 (94.9)	22,413 (94.1)	34,150 (95.5)	<0.001*
	Yes	3,020 (5.1)	1,394 (5.9)	1,626 (4.5)	
Emergency CS	No	51,559 (86.5)	20,846 (87.6)	30,713 (85.8)	<0.001*
	Yes	8,024 (13.5)	2,961 (12.4)	5,063 (14.2)	
Preterm	No	51,579 (86.6)	21,740 (91.3)	29,839 (83.4)	<0.001*
	Yes	8,004 (13.4)	2,067 (8.7)	5,937 (16.6)	
LBW	No	46,997 (86.2)	20,540 (87.4)	26,457 (85.3)	<0.001*
	Yes	7,524 (13.8)	2,949 (12.6)	4,575 (14.7)	
Sex of baby	Boy	32,132 (54.1)	12,771 (53.9)	19,361 (54.2)	0.391
	Girl	27,288 (45.9)	10,940 (46.1)	16,348 (45.8)	

For peer review only

Table 2. Binary logistic regression for full sample and GLMM analysis correcting for clustering (by hospital) displaying adjusted odds ratios

Chi2-test for group differences. n=54,919.

					Generalized Linear Mixed Method (GLMM)	
		Control N(%)	Intervention N(%)	P-value	aOR (95% CI)**	Intra-class correlation (ICC)
**Patient satisfaction**						
Are you satisfied with services and would you recommend a friend?	No	9070 (42.1)	12694 (38.0)		Ref	
	Yes	12485 (57.9)	20670 (62.0)	0.00	1.66 (1.59-1.73)	0.275
**Patient experience**						
Were you adequately informed by the care provider about examinations, actions and decisions taken for your care?	No	5021 (23.4)	6437 (19.3)		Ref	
	Yes	16439 (76.6)	26882 (80.7)	0.00	0.55 (0.53-0.58)	0.507
Are you satisfied with education/information?	No	12227 (56.7)	16922 (50.7)		Ref	

For peer review only - http://bmjopen.bmj.com/site/about/guidelines.xhtml

	Yes	9328 (43.3)	16422 (49.3)	0.00	1.34 (1.29-1.40)	0.167
Are you satisfied with level of privacy?	No	10433 (48.4)	16350 (49.0)		Ref	
	Yes	11122 (51.6)	17014 (51.0)	0.167	1.14 (1.09-1.18)	0.136
Were you or your newborn physically, verbally or sexually abused during labor or childbirth or after birth?	No	21308 (98.9)	33149 (99.4)		Ref	
	Yes	247 (1.1)	215 (0.6)	0.00	0.42 (0.34-0.51)	0.082
Were you treated with respect and was your dignity preserved during your stay at the hospital?	No	275 (1.3)	290 (0.9)		Ref	
	Yes	21280 (98.7)	33074 (99.1)	0.00	1.81 (1.52-2.16)	0.063
Did you have a companion of your choice during labor and child birth?	No	17837 (83.1)	26991 (81.0)		Ref	
	Yes	3623 (16.9)	6328 (19.0)	0.00	0.27 (0.25-0.29)	0.687

*Binary logistic regression models adjusted for caste/ethnicity and education level

** GLMM models adjusted for clustering (by hospital) and caste/ethnicity and education level

For peer review only - http://bmjopen.bmj.com/site/about/guidelines.xhtml

For peer review only

For peer review only - http://bmjopen.bmj.com/site/about/guidelines.xhtml

Figure legends

Figure 1. Stepped-wedge design and timeline of the NePeriQIP trial

The period within bold lines represents the baseline period. The shaded period constitutes the intervention period. Each wedge contains three hospitals (clusters).

Figure 2. Participants flow diagram

For peer review only

For peer review only - http://bmjopen.bmj.com/site/about/guidelines.xhtml

**Study month**	1	2	3	4	5	6	7	8	9	10	11	12	13	14	15	16	17	18
**Wedge 1**																		
**Wedge 2**																		
**Wedge 3**																		
**Wedge 4**																		
	Baseline			Step 1			Step 2			Step 3			Step 4					

209x297mm (300 x 300 DPI)

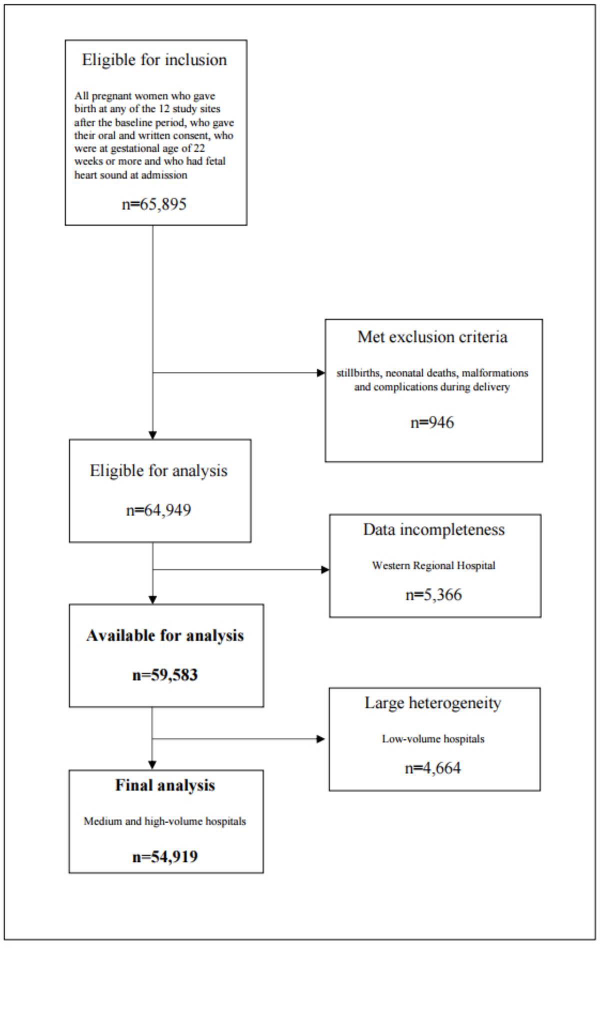

209x296mm (96 x 96 DPI)

Table S1. Binary logistic regression of patient satisfaction and quality measures of experience of care, by hospital. Adjusted for caste/ethnicity and education level (n=54,919).

		Satisfaction			Informed			Educated			Privacy			Abuse			Respect			Companion		
**Hospital**		Yes N(%)	No N(%)	Adj OR (95% CI)	Yes N(%)	No N(%)	Adj OR (95% CI)	Yes N(%)	No N(%)	Adj OR (95% CI)	Yes N(%)	No N(%)	Adj OR (95% CI)	Yes N(%)	No N(%)	AdjOR (95% CI)	Yes N(%)	No N(%)	AdjOR (95% CI)	Yes N(%)	No N(%)	AdjOR (95% CI)
Mid Western Region	Control	194 (66.4)	98 (33.6)	Ref	284 (97.3)	8 (2.7)	Ref	27 (9.2)	265 (90.8)	Ref	20 (6.8)	272 (93.2)	Ref	1 (0.3)	291 (99.7)	Ref	290 (99.3)	2 (0.7)	Ref	2 (0.7)	290 (99.3)	Ref
	Intervention	2318 (54.1)	1966 (45.9)	0.59 (0.46-0.76)	4252 (99.3)	30 (0.7)	3.99 (1.81-8.80)	1525 (35.6)	2759 (64.4)	5.45 (3.65-8.14)	1171 (27.3)	3113 (72.7)	5.12 (3.24-8.11)	42 (1.0)	4242 (99.0)	2.81 (0.39-20.5)	4259 (99.4)	25 (0.6)	1.14 (0.27-4.84)	12 (0.3)	4270 (99.7)	0.38 (0.08-1.70)
Bharatpur	Control	751 (23.7)	2418 (76.3)	Ref	2945 (93.7)	198 (6.3)	Ref	863 (27.2)	2306 (72.8)	Ref	871 (27.5)	2298 (72.5)	Ref	102 (3.2)	3067 (96.8)	Ref	3057 (96.5)	112 (3.5)	Ref	1285 (40.9)	1858 (59.1)	Ref
	Intervention	4971 (52.3)	4527 (47.7)	3.57 (3.26-3.91)	9224 (97.2)	265 (2.8)	2.34 (1.94-2.83)	5708 (60.1)	3790 (39.9)	4.07 (3.72-4.45)	5091 (53.6)	4407 (46.4)	3.08 (2.82-3.37)	63 (0.7)	9435 (99.3)	0.20 (0.15-0.27)	9421 (99.2)	77 (0.8)	4.54 (3.39-6.09)	174 (1.8)	9315 (98.2)	0.03 (0.02-0.03)
Seti Zonal	Control	919 (41.3)	1304 (58.7)	Ref	1959 (88.2)	261 (11.8)	Ref	421 (18.9)	1802 (81.1)	Ref	867 (39.0)	1356 (61.0)	Ref	37 (1.7)	2186 (98.3)	Ref	2180 (98.1)	43 (1.9)	Ref	1623 (73.1)	597 (26.9)	Ref
	Intervention	3778 (58.9)	2636 (41.1)	2.07 (1.87-2.28)	6107 (95.3)	303 (4.7)	2.74 (2.30-3.26)	3056 (47.6)	3358 (52.4)	3.93 (3.50-4.42)	3658 (57.0)	2756 (43.0)	2.11 (1.92-2.33)	35 (0.5)	6379 (99.5)	0.32 (0.20-0.50)	6331 (98.7)	83 (1.3)	1.53 (1.05-2.21)	5814 (90.7)	596 (9.3)	3.59 (3.16-4.07)
Koshi Zonal	Control	2777 (85.6)	468 (14.4)	Ref	2190 (67.8)	1040 (32.2)	Ref	2327 (71.7)	918 (28.3)	Ref	2606 (80.3)	639 (19.7)	Ref	9 (0.3)	3236 (99.7)	Ref	3219 (99.2)	26 (0.8)	Ref	495 (15.3)	2735 (84.7)	Ref
	Intervention	3886 (90.7)	398 (9.3)	1.83 (1.57-2.12)	3148 (73.7)	1124 (26.3)	1.33 (1.20-1.47)	2841 (66.3)	1443 (33.7)	0.82 (0.74-0.90)	2859 (66.7)	1425 (33.3)	0.53 (0.48-0.60)	32 (0.7)	4252 (99.3)	2.59 (1.21-5.53)	4252 (99.3)	32 (0.7)	0.99 (0.58-1.69)	256 (6.0)	4016 (94.0)	0.36 (0.31-0.43)

For peer review only

Rapti Subregional	Control	785 (31.8)	1686 (68.2)	Ref	995 (40.3)	1473 (59.7)	Ref	569 (23.0)	1902 (77.0)	Ref	1108 (44.8)	1363 (55.2)	Ref	17 (0.7)	2454 (99.3)	Ref	2424 (98.1)	47 (1.9)	Ref	22 (0.9)	2446 (99.1)	Ref
	Intervention	271 (15.5)	1473 (84.5)	0.39 (0.34-0.46)	266 (15.3)	1478 (84.7)	0.27 (0.23-0.31)	96 (5.5)	1648 (94.5)	0.19 (0.15-0.24)	875 (50.2)	869 (49.8)	1.23 (1.09-1.39)	17 (1.0)	1727 (99.0)	1.42 (0.72-2.80)	1732 (99.3)	12 (0.7)	2.86 (1.51-5.42)	29 (1.7)	1715 (98.3)	1.97 (1.13-3.46)
Lumbini zonal	Control	5512 (87.5)	791 (12.5)	Ref	4445 (71.0)	1813 (29.0)	Ref	4032 (64.0)	2271 (36.0)	Ref	4378 (69.5)	1925 (30.5)	Ref	73 (1.2)	6230 (98.8)	Ref	6268 (99.4)	35 (0.6)	Ref	183 (2.9)	6075 (97.1)	Ref
	Intervention	4242 (88.3)	564 (11.7)	1.05 (0.94-1.18)	2739 (57.1)	2058 (42.9)	0.54 (0.50-0.58)	2830 (58.9)	1976 (41.1)	0.80 (0.74-0.87)	2987 (62.2)	1819 (37.8)	0.72 (0.66-0.78)	17 (0.4)	4789 (99.6)	0.31 (0.18-0.52)	4764 (99.1)	42 (0.9)	0.63 (0.40-0.98)	42 (0.9)	4755 (99.1)	0.30 (0.21-0.42)
Bheri Zonal	Control	1547 (40.2)	2305 (59.8)	Ref	3621 (94.1)	228 (5.9)	Ref	1089 (28.3)	2763 (71.7)	Ref	1272 (33.0)	2580 (67.0)	Ref	8 (0.2)	3844 (99.8)	Ref	3842 (99.7)	10 (0.3)	Ref	13 (0.3)	3836 (99.7)	Ref
	Intervention	1204 (51.6)	1130 (48.4)	1.62 (1.46-1.80)	1146 (49.3)	1179 (50.7)	0.61 (0.05-0.07)	386 (16.5)	1948 (83.5)	0.51 (0.45-0.58)	373 (16.0)	1961 (84.0)	0.39 (0.34-0.45)	9 (0.4)	2325 (99.6)	1.88 (0.72-4.87)	2315 (99.2)	19 (0.8)	0.32 (0.15-0.69)	1 (0.0)	2324 (100.0)	0.13 (0.02-0.96)
All	Control	12485 (57.9)	9070 (42.1)	Ref	16439 (76.6)	5021 (23.4)	Ref	9328 (43.3)	12227 (56.7)	Ref	11122 (51.6)	10433 (48.4)	Ref	247 (1.1)	21308 (98.9)	Ref	21280 (98.7)	275 (1.3)	Ref	3623 (16.9)	17837 (83.1)	Ref
	Intervention	20670 (62.0)	12694 (38.0)	1.20 (1.16-1.25)	26882 (80.7)	6437 (19.3)	1.26 (1.21-1.31)	16442 (49.3)	16922 (50.7)	1.28 (1.24-1.33)	17014 (51.0)	16350 (49.0)	0.98 (0.95-1.01)	215 (0.6)	33149 (99.4)	0.55 (0.46-0.66)	33074 (99.1)	290 (0.9)	1.49 (1.27-1.76)	6328 (19.0)	26991 (81.0)	1.12 (1.07-1.17)

For peer review only

For peer review only

CONSORT 2010 checklist of information to include when reporting a randomised trial*

Section/Topic	Item No	Checklist item	Reported on page No
**Title and abstract**			
	1a	Identification as a randomised trial in the title	1
	1b	Structured summary of trial design, methods, results, and conclusions (for specific guidance see CONSORT for abstracts)	2
**Introduction**			
Background and objectives	2a	Scientific background and explanation of rationale	4
	2b	Specific objectives or hypotheses	5
**Methods**			
Trial design	3a	Description of trial design (such as parallel, factorial) including allocation ratio	5
	3b	Important changes to methods after trial commencement (such as eligibility criteria), with reasons	N/A
Participants	4a	Eligibility criteria for participants	6
	4b	Settings and locations where the data were collected	5-6
Interventions	5	The interventions for each group with sufficient details to allow replication, including how and when they were actually administered	6
Outcomes	6a	Completely defined pre-specified primary and secondary outcome measures, including how and when they were assessed	7-9
	6b	Any changes to trial outcomes after the trial commenced, with reasons	N/A
Sample size	7a	How sample size was determined	9
	7b	When applicable, explanation of any interim analyses and stopping guidelines	N/A
Randomisation:			
Sequence generation	8a	Method used to generate the random allocation sequence	N/A
	8b	Type of randomisation; details of any restriction (such as blocking and block size)	N/A
Allocation concealment mechanism	9	Mechanism used to implement the random allocation sequence (such as sequentially numbered containers), describing any steps taken to conceal the sequence until interventions were assigned	N/A
Implementation	10	Who generated the random allocation sequence, who enrolled participants, and who assigned participants to interventions	N/A
Blinding	11a	If done, who was blinded after assignment to interventions (for example, participants, care providers, those	N/A

For peer review only

		assessing outcomes) and how	
	11b	If relevant, description of the similarity of interventions	N/A
Statistical methods	12a	Statistical methods used to compare groups for primary and secondary outcomes	9
	12b	Methods for additional analyses, such as subgroup analyses and adjusted analyses	9
**Results**			
Participant flow (a diagram is strongly recommended)	13a	For each group, the numbers of participants who were randomly assigned, received intended treatment, and were analysed for the primary outcome	10
	13b	For each group, losses and exclusions after randomisation, together with reasons	10
Recruitment	14a	Dates defining the periods of recruitment and follow-up	7
	14b	Why the trial ended or was stopped	N/A
Baseline data	15	A table showing baseline demographic and clinical characteristics for each group	17
Numbers analysed	16	For each group, number of participants (denominator) included in each analysis and whether the analysis was by original assigned groups	10-11
Outcomes and estimation	17a	For each primary and secondary outcome, results for each group, and the estimated effect size and its precision (such as 95% confidence interval)	10-11
	17b	For binary outcomes, presentation of both absolute and relative effect sizes is recommended	10-11
Ancillary analyses	18	Results of any other analyses performed, including subgroup analyses and adjusted analyses, distinguishing pre-specified from exploratory	10-11
Harms	19	All important harms or unintended effects in each group (for specific guidance see CONSORT for harms)	N/A
**Discussion**			
Limitations	20	Trial limitations, addressing sources of potential bias, imprecision, and, if relevant, multiplicity of analyses	13
Generalisability	21	Generalisability (external validity, applicability) of the trial findings	13
Interpretation	22	Interpretation consistent with results, balancing benefits and harms, and considering other relevant evidence	11-13
**Other information**			
Registration	23	Registration number and name of trial registry	14
Protocol	24	Where the full trial protocol can be accessed, if available	14
Funding	25	Sources of funding and other support (such as supply of drugs), role of funders	14

*We strongly recommend reading this statement in conjunction with the CONSORT 2010 Explanation and Elaboration for important clarifications on all the items. If relevant, we also recommend reading CONSORT extensions for cluster randomised trials, non-inferiority and equivalence trials, non-pharmacological treatments, herbal interventions, and pragmatic trials. Additional extensions are forthcoming: for those and for up to date references relevant to this checklist, see www.consort-statement.org.

For peer review only - http://bmjopen.bmj.com/site/about/guidelines.xhtml
